# Computational discovery of molecular C_60_ encapsulants with an evolutionary algorithm

**DOI:** 10.1038/s42004-020-0255-8

**Published:** 2020-01-22

**Authors:** Marcin Miklitz, Lukas Turcani, Rebecca L. Greenaway, Kim E. Jelfs

**Affiliations:** 1 Department of Chemistry, Molecular Sciences Research Hub, White City Campus, Imperial College London, Wood Lane, London, W12 0BZ UK; 2 Department of Chemistry and Materials Innovation Factory, University of Liverpool, 51 Oxford Street, Liverpool, L7 3NY UK

**Keywords:** Materials chemistry, Computational chemistry, Supramolecular chemistry, Molecular capsules

## Abstract

Computation is playing an increasing role in the discovery of materials, including supramolecular materials such as encapsulants. In this work, a function-led computational discovery using an evolutionary algorithm is used to find potential fullerene (C_60_) encapsulants within the chemical space of porous organic cages. We find that the promising host cages for C_60_ evolve over the simulations towards systems that share features such as the correct cavity size to host C_60_, planar tri-topic aldehyde building blocks with a small number of rotational bonds, di-topic amine linkers with functionality on adjacent carbon atoms, high structural symmetry, and strong complex binding affinity towards C_60_. The proposed cages are chemically feasible and similar to cages already present in the literature, helping to increase the likelihood of the future synthetic realisation of these predictions. The presented approach is generalisable and can be tailored to target a wide range of properties in molecular material systems.

## Abstract (web summary)

Computation is proving to be a powerful tool to guide the design of supramolecular cages. Here the authors use an evolutionary algorithm to find potential fullerene encapsulants within the chemical space of porous organic cages.

## Introduction

Arguably, the majority of cases of the discovery of new materials are dependent upon small changes to known systems based on chemical knowledge or are a result of a serendipitous discovery. However, computation is playing an increasing role in the rational design and discovery of new advanced materials^1^. For example, the high-throughput computational screening of existing and hypothetical compounds can facilitate identification of materials with optimal properties or help formulate structure-property relationships for future rational materials discovery^2,3^. High-throughput screens can be used to perform brute force searches of a large number of possible materials, accelerated by increasing computational power or machine learning, and covering much larger regions of phase space than can be reasonably accessed experimentally, even with automation.

Porous molecular materials are distinct from porous network materials such as zeolites, metal-organic frameworks (MOFs) and polymers, in that they are made up of discrete molecular units rather than having three-dimensional extended bonding^4,5^. Molecules can be porous in the solid-state through either extrinsic porosity, where the molecules are unable to pack efficiently to remove void space, or through intrinsic porosity, where the molecule itself has a persistent internal cavity. Examples of intrinsically porous molecules include calixarenes, cucubiturils and organic cages, and these systems are investigated for applications in molecular separation, encapsulation, catalysis, sensing, and as porous liquids^4^. Porous organic cages (POCs) are polycyclic molecules that have three-dimensional structures with three or more molecular windows^4^.

The discovery of new POCs consists of many challenges; first, after the successful synthesis of the required precursors for the systems, they must be combined to form the cage species, which is typically done via a one-pot reaction using dynamic covalent chemistry (DCC). The most common type of DCC reaction used to form cages is imine condensation. During this process, not every reaction will successfully form a cage, for example, in a recent high-throughput screening study only 42% of the reactions were successful^6^. Furthermore, not only does one need a successful reaction, but the reaction needs to form the molecule in the desired topology and for the molecule to be shape persistent if desired, retaining an internal cavity in the absence of solvent. The topology formed can be hard to predict a priori^7^, and, further, we recently found that of 63,472 hypothetical cages, built from a library of precursors with shape persistence in mind, only 28% were actually shape persistent^8^.

Computation can help guide the discovery of POCs, with calculations considering the thermodynamics and kinetic pathways of the assembly process able to help identify the expected topology of a given reaction^6,9,10^, and whether or not it is shape persistent^8,11^. We have also recently applied supervised machine learning to accelerate the prediction of shape persistence of a hypothetical cage assembly, making this accessible to the experimental community^8^. With a molecular structure, crystal structure prediction techniques can be used to unveil the most energetically favourable crystal packings^12^. While <200 POCs have so far been experimentally realised, in theory the search space for these systems is vast if all possible combinations of organic precursors for DCC reactions are considered. Of course, not all molecules are suitable building blocks for POCs, nor are the majority likely to be synthetically accessible, but this just creates an additional challenge in the sensible selection of precursors if one wants to truly consider a diverse range of possibilities, outside of what would be immediately available for synthetic screening.

It is not computationally feasible to analyse all combinations of organic building blocks as POCs for a given application. Recently, we developed open-source python-based software, called the supramolecular toolkit (*stk*), that allows the automated construction of different types of materials from precursor databases^13^. We recently showed that an extension of *stk* to include an evolutionary algorithm (EA) could be used to target specific structural features of POCs, such as high symmetry or a specific pore size, identifying not only promising targets, but also more general design rules to obtain a specific feature^14^. This has already led to the synthetic realisation of promising identified POCs^15^. EAs mimic evolutionary processes to solve global minimisation problems, with the evolutionary pressure for ‘survival of the fittest’ in our case being targeted towards a desired set of features in a molecular material. After calculating the quality of each of the candidates of a generation, the population is ‘evolved’ by performing modifications that mimic crossover and mutation in nature. EAs are used as efficient ways to sample chemical space for drug discovery^16^, and computational materials discovery^17^, including for porous network materials^18^. Here, rather than focusing on optimising a structural feature of the POCs, we focus for the first time on targeting a specific application of the cages, in this case the encapsulation of C_60_ within a cage when in solution. Through screening hundreds of possibilities and seeking to optimise the function of the cage, this differs to an approach for designing metal-organic cages to encapsulate materials by designing complementary geometries of the host^19^.

The application of fullerenes span over biomedicine^20^ and materials science^21^, for example in organic photovoltaic devices and superconductive materials^22,23^. A lot of effort has been applied to research into the selective binding of different species of fullerenes for the purification process^24,25^. The immobilisation of fullerenes in complexes enables controlled property tuning and selective formation of fullerene adducts^26,27^. Fullerenes can also act as templates and drive macrocycle formation towards desired supramolecular architectures^28^. The common mechanism of fullerene encapsulation is to maximise the non-specific van der Waals interactions between the host molecule that “wraps” itself around the fullerene, as in the “buckycatcher”^29^. There are multiple examples of bowl-shaped molecules binding with fullerene^30–32^, and some examples of metal-organic cage encapsulation^24,33^. POCs, however, have been mostly absent in fullerene host-guest supramolecular chemistry. The only two examples that have been proposed as possible fullerene hosts to our knowledge are a sandwich-like cage and a porphyrin cage (**COP-5**)^34,35^.

Here, our EA-based screening for POCs that are potential C_60_ encapsulants reveals specific cage targets that have common features such as a cavity diameter of ~10 Å and similar sized building blocks. We explore how to parameterise the EA and discuss how the approach could be applied to larger databases of potential cage building blocks, or targeted at other encapsulants or molecular materials with desired properties in the future.

## Results

### The database of assembled cages

A small custom database of precursors, 43 tri-topic (**Tri**) aldehydes and 90 di-topic (**Di**) amines (see Supplementary Figs. 1–4), was used to reduce the vast chemical space of possible precursors. This precursor database, when combined in every possible combination in a single topology, corresponds to 3870 imine cages. Here, we only consider cages assembled in a [4 + 6] reaction of four aldehydes and six diamines into a **Tri**^**4**^**Di**^**6**^ topology that relates to a tetrahedron, using the nomenclature introduced by Santolini et al.^9^. The trialdehydes are hereafter referred to as ‘nodes’ and diamines as ‘linkers’, based on their positioning on the template geometry in the cage assembly process (trialdehydes on the vertices and diamines on the edges). These precursors were either selected from previously reported organic cages, or are molecules that we deemed synthetically viable and reasonable precursors for cage synthesis, but have not been previously reported. The same set of precursors was used in our previous study using machine learning to predict shape persistence^8^. This database is intentionally limited in size to allow quick screening for the purpose of the fitness function (FF) parameterisation. To simplify the description of the derived POCs and C_60_@POC complexes, a generated POC is simply referenced to as the “cage” and the corresponding C_60_@POC complex as the “complex”. The final population of cages were assigned code names of type **CX**, where **X** is a number in ascending order and **C1** is a cage of highest fitness value. Lastly, “**CX** complex” corresponds to the C_60_@**CX** complex.

**Fig. 1:**
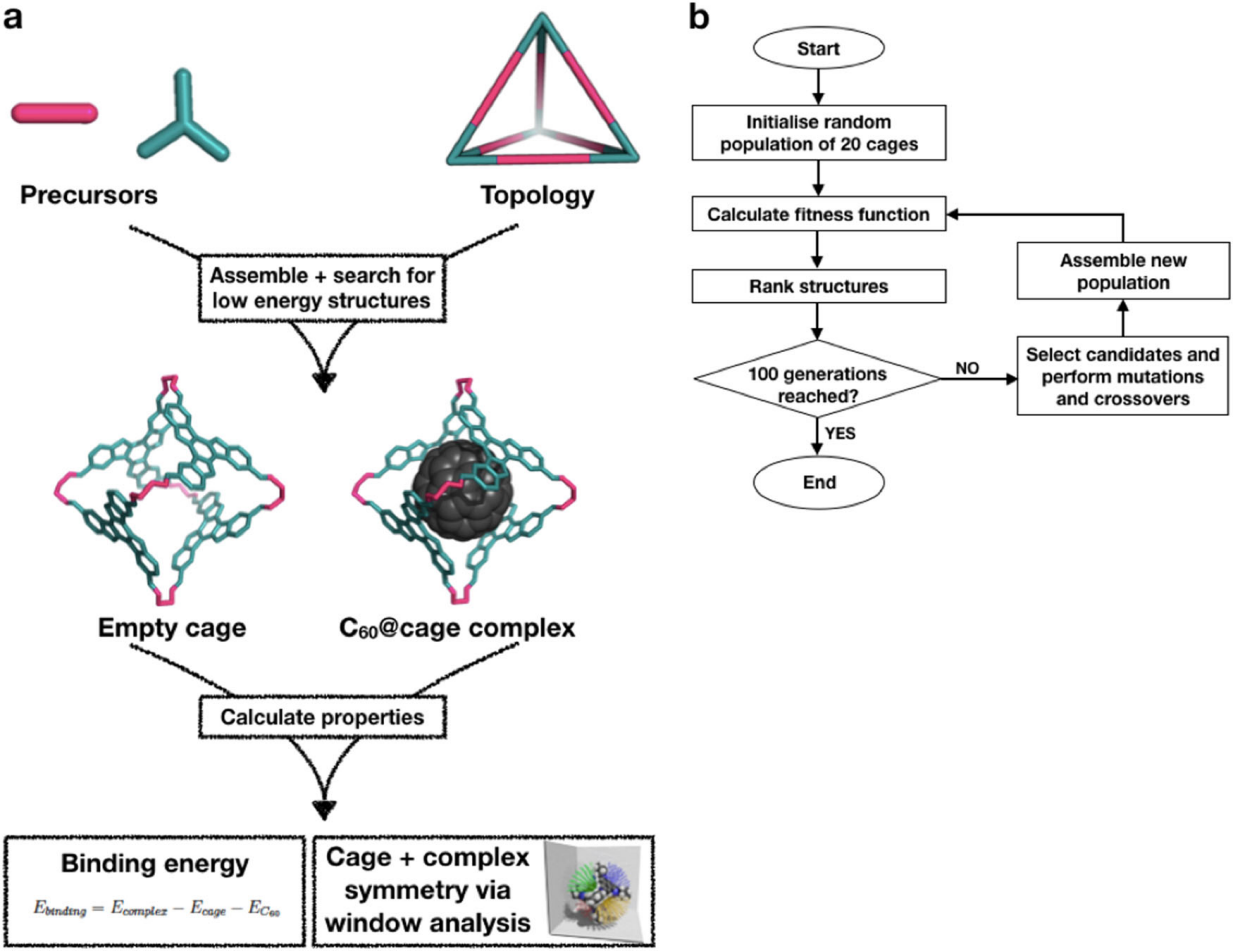
An overview of the computational workflow. **a** A summary of the cage assembly and property assessment process; **b** Overall workflow for the evolutionary algorithm.

**Fig. 2:**
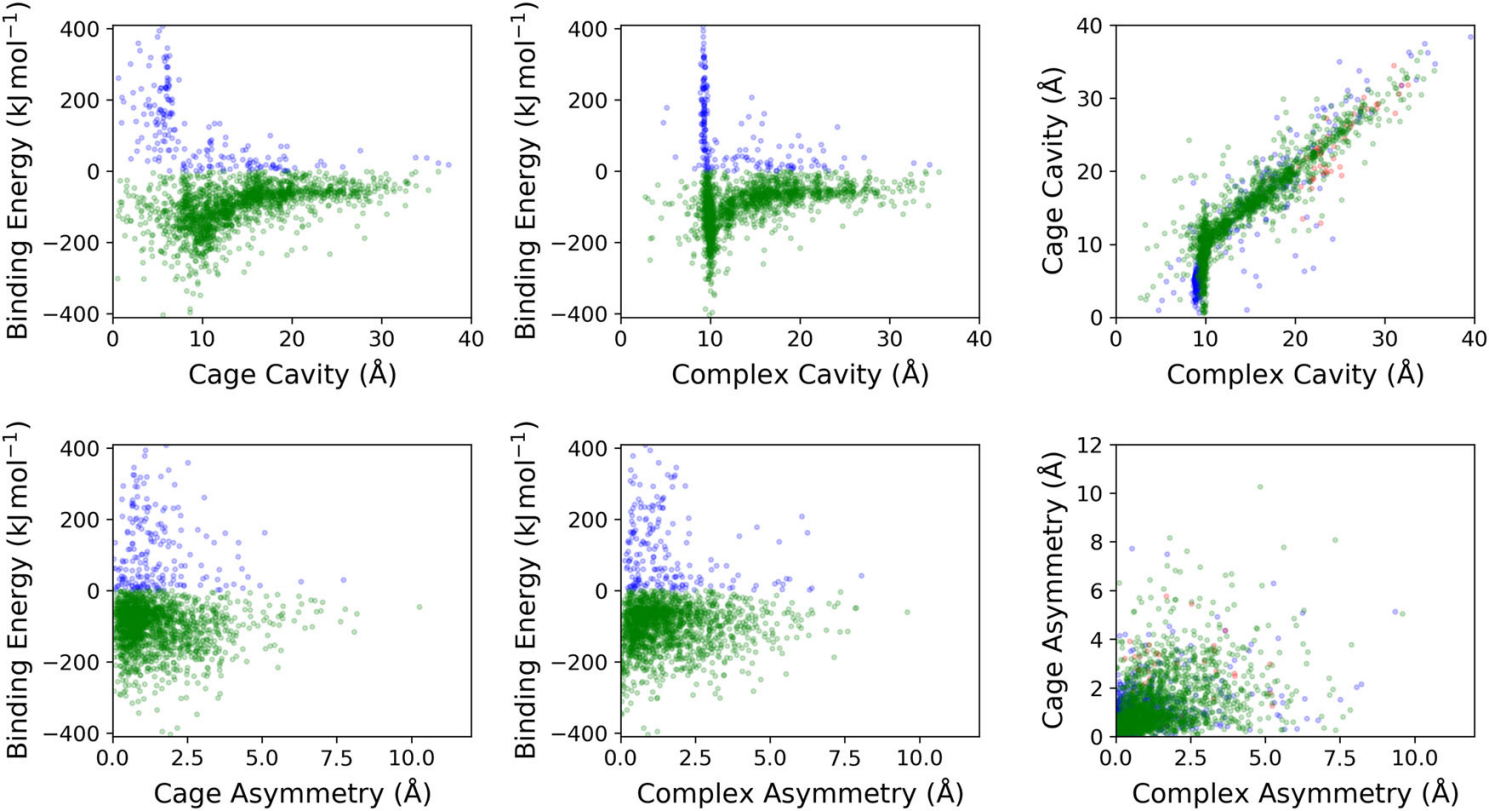
The properties of the assembled POCs and C_60_ complexes. The complexes were divided into three sets: those with binding energies >0 kJ mol^−1^ (blue), those with binding energies between  −404 and 0 kJ mol^−1^ (green), and those with binding energies well below  −404 kJ mol^−1^ (red).

An overview of the assembly and property calculation process for a cage is shown in Fig. 1a. The calculated properties of the geometry optimised cages and their corresponding complexes are shown in Fig. 2 and Supplementary Figs. 5–8. The pairs of POCs and their complexes were divided into three groups: the complexes that have C_60_ binding energies greater than 0 kJ mol^−1^ (repulsive interaction); complexes with binding energies within the range of  −404 and 0 kJ mol^−1^; and complexes with binding energies well below  −404 kJ mol^−1^. The last set, coloured red in the graphs, was notable as these forcefield binding energies seemed unreasonable. Grimme et al. reported binding energies of  −770 and  −606 kJ mol^−1^ for a hypothetical multi-shell “hyperfullerene” complex (C_60_@C_240_), where these values can be seen as a physical limit of the C_60_ interactions with a potential host^36^. We note that these simulated binding energies will be of considerably greater magnitude than any experimentally measured values due to the absence of solvent in our simulations. However, in the set of complexes coloured red, the binding energies are in the range of a few thousands of  −kJ mol^−1^. Additionally, these seem to aggregate around certain values and are observed for POCs containing larger cavities, in the region of 20–30 Å (the C_60_ diameter is  ~10 Å). These were inspected and determined to have unreasonable geometries with the POC structures ‘stuck’ in chemically infeasible geometries, for example with an unusual orientation of hydrogens. It is believed this is a systematic error (as there is a similarity of binding energy values between groups of cages) and a false result, thus these POCs were disregarded.

Those complexes with attractive binding energies between 0 and  −404 kJ mol^−1^ in Fig. 2 have a particular focus around cavity sizes of  ~10 Å, more so in fact than in the isolated POC molecules. This is the approximate size of the C_60_ molecule and reflects the fact that many of the POCs have expanded their intrinsic cavity to form one of the correct size for hosting C_60_. This is the reason for a large set of complexes with positive binding energies (blue points); the energy penalty of adapting to fit C_60_ is far greater than the benefits of the C_60_ presence. The green set of cages with favourable binding energies is the target group of complexes for the EA.

### FF parameterisation

The FF is used to calculate the performance of a cage as a C_60_ encapsulant during a run in our EA. The FF parameterisation was first performed on the database of all assembled cages and their complexes. For the purpose of the FF parameterisation, the complete database of 3870 cages and their C_60_ complexes were generated and the C_60_ binding energy in the complex at the forcefield level (*E*_binding_) and the asymmetry of the cage extracted from the complex (*A*_complex_) were calculated. The geometry optimisation process is the bottleneck of the EA calculations in this work, and the database of pre-assembled and geometry optimised cages resulting from all combinations of precursors allowed for a quick screening of a range of constants and powers for the FF to find the right parameters. The FF had the form:

1 $${\mathrm{FF}}={(a{E}_{\mathrm{binding}}^{b}+c{A}_{\mathrm{complex}}^{d})}^{-1}$$

and *a* and *c* constants were screened for values between 1 and 5, in increments of 1, for all combinations. The *b* and *d* exponents for all combinations of values in the range of 0–5 were considered in increments of 0.25.

**Fig. 3:**
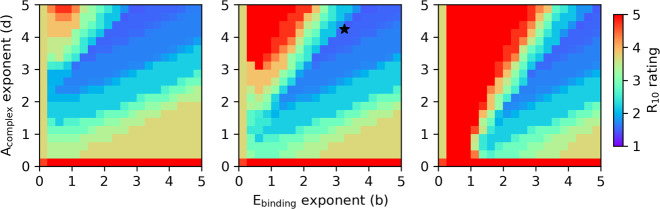
The heat maps of the fitness function parameterisation process. The plots from left to right are for the *a* and *c* constants of 5:1, 1:1 and 1:5, respectively. The *x* and *y* axes correspond to the screened *b* and *c* exponents. The heat map scale shows the R_10_ score for the ten cages with highest fitness. The lower the R_10_ score, the higher quality of the set. The *a*, *c*, *b*, *d* parameter combination (1, 1, 3.25 and 4.25, respectively) that gave the lowest *R*_10_ score of 1.480 is marked with a star.

During the FF calculation, collapsed cages that have lost their internal cavity are discarded, allowing us to focus on shape-persistent, symmetric molecules as potential C_60_ encapsulants. It is facile to identify these systems, as these will not have windows whose diameter can be determined by *pywindow*. We found that 37% of the POCs were discarded because either the empty cage, complex, or both, failed the asymmetry criteria, with the majority of collapsed cases coming from the empty systems (21%). If we then factor in wanting to have a favourable interaction energy, then 44% of cages fit that criteria. For all 2432 of these cages, the FF was calculated with the full set of parameters, equating to 9261 different setups, 21 different ratios of constants *a* and *c* and for each of these, a heat map was generated with 441 combinations of exponents *b* and *d*. For each *a*, *b*, *c*, *d* parameter combination, the *R*_10_ score was calculated with Eq. (4) for the set of ten cages with the highest fitness value. The *R*_10_ score gives the relative quality of the set of ten best cages in respect to other sets of parameters for the FF. The results presented in Fig. 3 are for the set of constants *a*:*c* for 1:1 (middle), 5:1 (left) and 1:5 (right) ratios, which show the general trend observed for combinations of *a* and *c*. The lowest *R*_10_ score corresponds to the optimal set of parameters. The lowest *R*_10_ value was 1.480 and was identified for 126 different sets of parameters. The simplest set of parameters, where the sum of *a*, *c*, *b* and *d* was smallest, was then considered. The identified set of parameters was *a* = 1, *b* = 3.25, *c* = 1 and *d* = 4.25.

We can learn lessons from this parameterisation procedure that can be used in other studies in the future that seek an optimal set of weightings for an EA FF to search large databases for molecular materials. Firstly, a rigorous approach would involve generating a random subset of all potential solutions, and then a parameterisation performed as we have done here, before applying that parameterisation to a search of the full database. However, we can also see that if you already have components in the FF, then the setup can be generalised to add additional related components, for example cage and complex asymmetry here. What we learnt from our extensive parameterisation, was that in the end the weightings of the components essentially matched what we would have expected from chemical intuition. Thus, for a system where there is familiarity with the importance of the components, the parameterisation step could be skipped. Finally, while the exponent values of 3.25 and 4.25 were found to give the best rankings in this case, we would suggest that it would also be sufficient, and simpler, to use exponents of 1 in future studies.

### The evolutionary algorithm calculations

A flowchart summarising the key steps in the EA is shown in Fig. 1b. With the FF parameterised, five separate EA calculations were performed on the database of precursors presented. The final goal was to find excellent POC candidates for C_60_ encapsulation and the FF had the final form:

2 $${\mathrm{FF}}={({E}_{\mathrm{binding}}^{3.25}+0.5{A}_{\mathrm{complex}}^{4.25}+0.5{A}_{\mathrm{cage}}^{4.25})}^{-1}$$

At this point we introduced the new feature of *A*_cage_, the asymmetry of the isolated cage. While we simplified the initial parameterisation to only have two components (*E*_binding_ and *A*_complex_) to make it manageable, we added this additional feature here as discussion with synthetic chemists had suggested that higher symmetry isolated cage molecules should have a higher likelihood of being synthesised. To weight the binding energy equally to the asymmetry consideration, the asymmetry-related parameters were given half weights (constants of 0.5), so that the sum of the constants equals that of the binding energy. Each EA calculation was run for 100 generations, with a population size of 20.

**Fig. 4:**
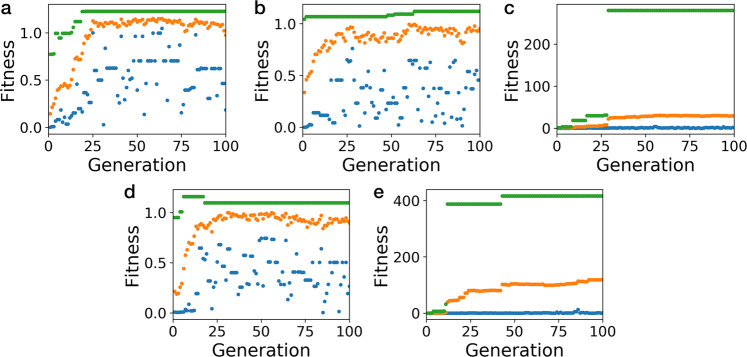
The evolution of the fitness values in the five EA calculations. The lowest (blue), highest (green) and average (orange) fitness function in each generation of 20 members is shown for runs 1–5 (**a**–**e**). The fitness value is with respect to a given population, therefore the scales on each of the graphs have different magnitude and can not be compared between separate EA calculations.

The evolution of the FF in each run is shown in Fig. 4. We can see that in all cases, the mean fitness value quickly increases and then converges. In most cases, convergence occurs relatively quickly, after  ~25 generations. Supplementary Fig. 9 shows the breakdown of the absolute values of the three components of the FF; the binding energy, complex asymmetry and cage asymmetry. These show that although the binding energy converges quickly, with essentially no change after 20 generations, the asymmetry values fluctuate more, with an overall trend to lower mean values for the asymmetry (i.e. more symmetric structures), which is generally converged by about 50 generations. This suggests that finding high binding energy complexes is easier than finding symmetric cages and assemblies. As more symmetric cage systems stand a greater likelihood of being synthetically realised, it is important to use the longer runs to fine tune these features. These findings emphasise a common feature in computational materials discovery programs—that it is comparatively easy to find materials with good property performance, but harder for the materials to also be experimentally viable.

**Fig. 5:**
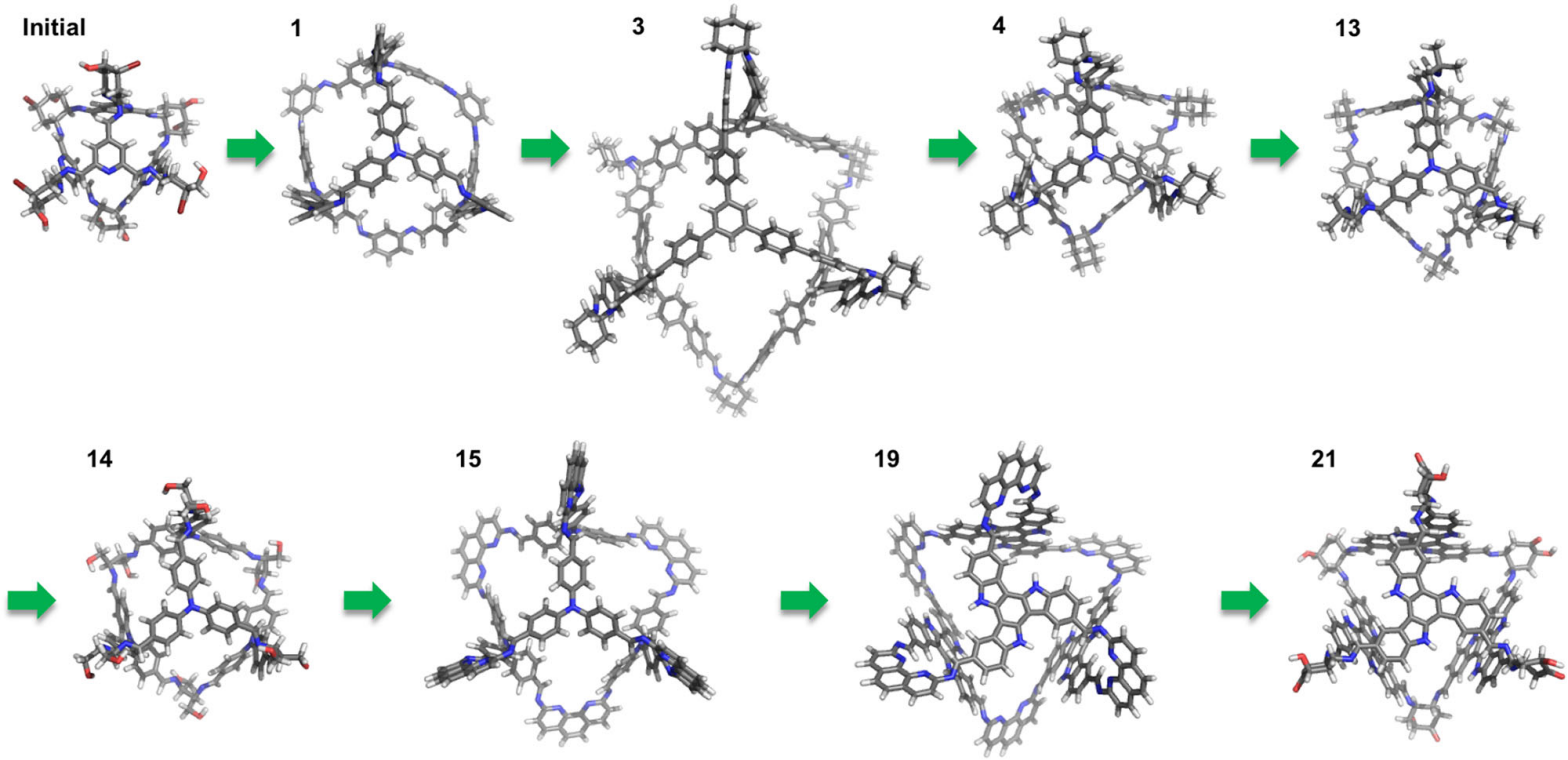
The evolution of a high performing C_60_ cage encapsulant. The top performing candidates from run 1 at different stages of the evolution. The generation number where each structure becomes the top performing is labelled.

To rank the cages from all five EA calculations, the results were combined and reweighted with respect to the same FF from Eq. (2). The combined results consisted of 53 unique cages (duplicates were discarded) and their complexes. Figure 5 shows how the top scoring cage evolves over the generations for run 1 and Supplementary Figs. 10–13 show the same for the other runs. While each run is different, and obviously seeing only the top candidate only provides so much information, it can be seen that the cages typically start with a cavity that is too small or too large for C_60_, alternative sizes are then trialled, but once there is a top candidate with approximately the correct size for C_60_, the cavity size of the top candidate essentially no longer changes, but rather there are only changes to the exact chemical composition of the components of the cage, as the EA seeks to maximise the FF. We note that the top couple of cages can swap, with a specific candidate no longer being ranked top before returning to top; this is due to the precise ranking depending on the composition of the entire population in our normalisation process.

To examine how chemically diverse the building blocks of the cages were, and how this evolved over the course of the EA, we calculated the mean Dice similarity of Morgan fingerprints of radius 2 between all unique pairs within each generation. As shown in Supplementary Fig. 19, the mean Dice similarity across random building blocks at initialisation is approximately 0.34. In all runs, the mean Dice similarity increases to between 0.4 and 0.5 over the course of the run. This makes sense as, for example, some of the building blocks that are too large or too small to form the correct size pore are not selected, resulting in populations that occupy a smaller region of chemical space as the EA continues. However, as features such as external functionalisation of the cage are not under evolutionary pressure, there could be significant differences in those regions of the cage building blocks. The (small) range of different values across the five runs also indicates that different final populations are found, even if many of the top candidates are the same.

We further carried out a structural analysis of the cages over each of the EA runs, calculating the average percentage of double bonds and rotatable bonds in the cages at each generation (see Supplementary Figs. 14–18). We found that the runs typically converge to cages having an average of 5–15% of their bonds being classed as rotatable, with the linker typically having a greater degree of rotatable bonds, and just below 10% double bonds in the molecule for the linker and almost 30% double bonds for the node. These features can be considered as design rules for molecules that encapsulate C_60_. To aid analysis of convergence in the future, tools which identify the salient features of building blocks with regard to the pore, and compare those only, would provide a more accurate picture of convergence.

**Fig. 6:**
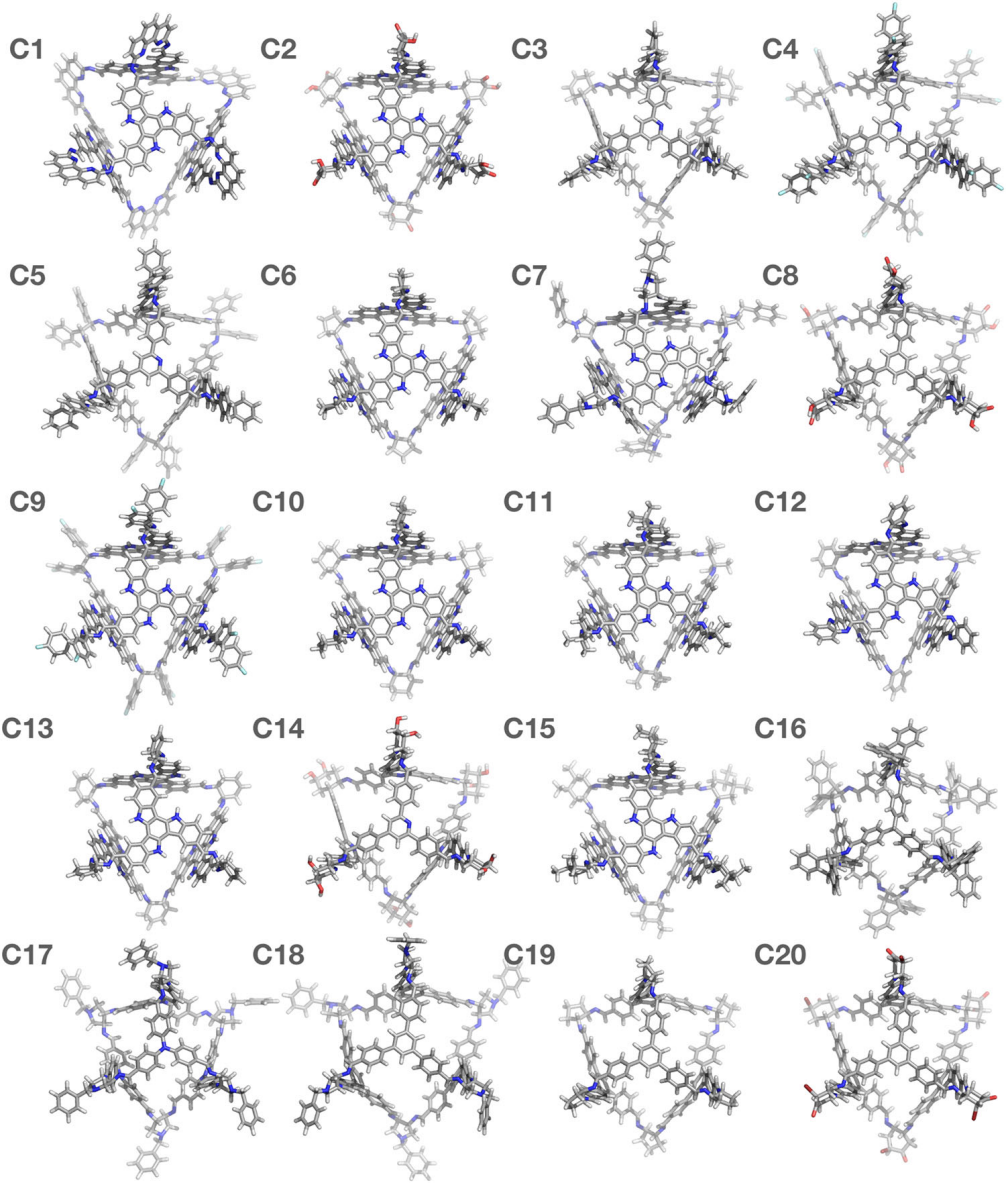
The 20 best cages found for C_60_ encapsulation. The 20 cages with the best fitness values from the combined results of the five EA calculations sorted from best (**C1**) to worst (**C20**). The geometry optimised structures of the empty POCs are presented.

**Fig. 7:**
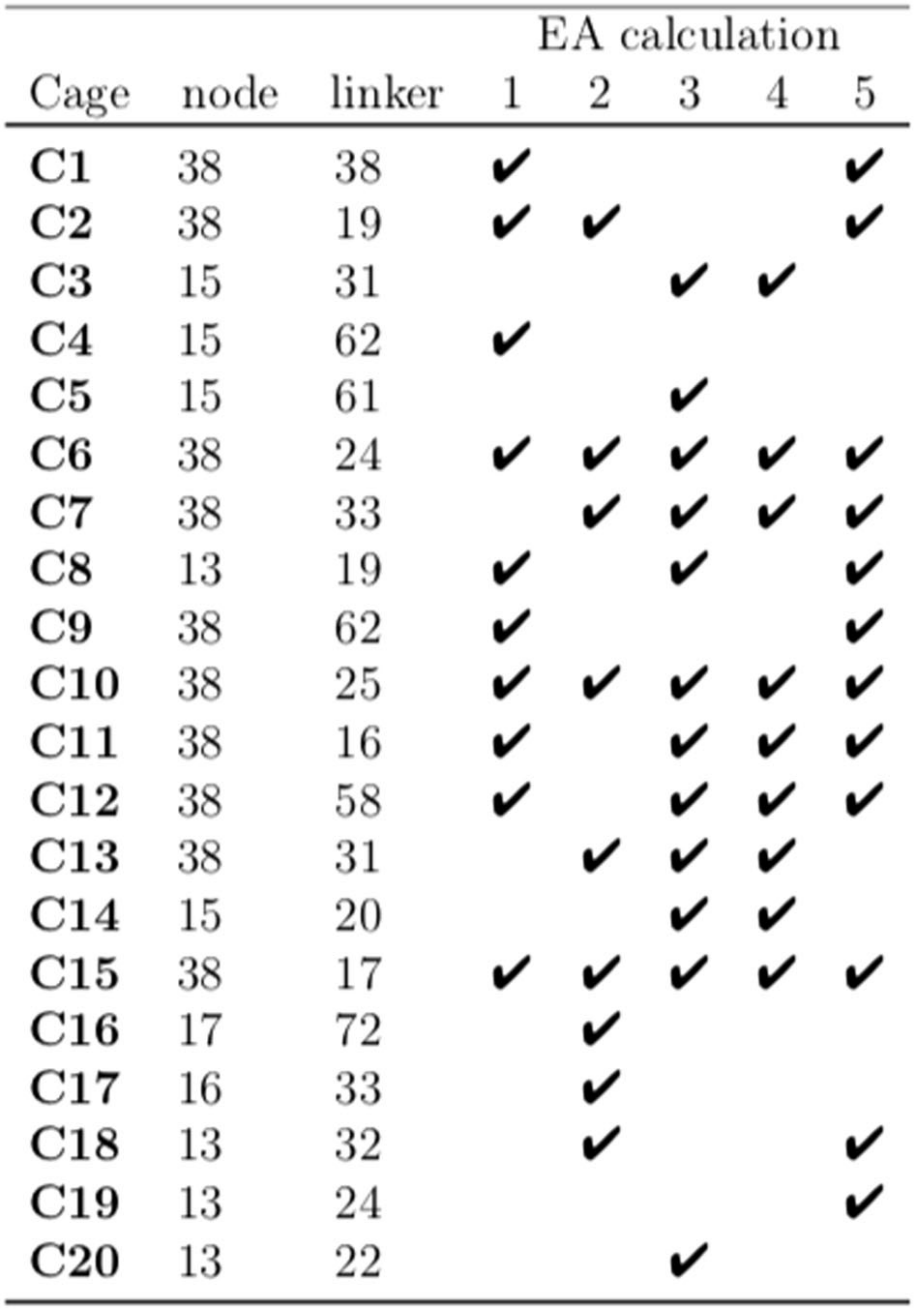
The aldehyde nodes and amine linkers used to assemble the final population of cages. The node and linker index numbers correspond to Supplementary Figs. 1–4. The specific EA calculations in which each cage was a member of the 100th generation are marked with a tick sign.

The 20 cages with the best fitness values are presented in Fig. 6. In Fig. 7, the nodes and linkers that the 20 cages were assembled from are listed. In addition, each EA run that identified a given cage is marked with a tick sign. The fact that many of these cages were identified multiple times shows the effectiveness of the constructed FF and that the screening of the databases is quick and broad.

The nodes in the 20 best cages share similar features. They are planar, have a small number of rotatable bonds, and have a high number of aromatic rings. They are also very similar in size. The **node38** has a spherical diameter of 16.1 Å, and **node15** and **node13** have diameters of 16.8 and 17.0 Å, whereas **node17** and **node16** are slightly smaller and have diameters of 14.1 and 14.4 Å, respectively. The **linker38** in **C1** is the most distinct from the set of linkers, as the separation of the nitrogens between neighbouring imine bonds is 6.9 Å. All the other linkers have the amine functionality on neighbouring carbons, resulting in the spacing between nitrogens in imine bond pairs in a range of 3.0–3.4 Å. While the linker in **C1** is comparatively larger, this does not result in a larger cavity diameter in comparison with the rest of the cages.

In Table 1, the re-scaled fitness values for the combined results of the five EA calculations and the unscaled parameters are presented. The cages have relatively high magnitude binding energies, between  −160 and  −270 kJ mol^−1^. The asymmetry for both the empty cage and the cage complex are also in the lower range of values present in the database, so the final assemblies and their corresponding POCs are all very symmetrical. The POCs have cavity diameters between 9.7 and 10.6 Å, all close in size to the C_60_ diameter (~10 Å). This is despite the fact that the cavity diameter of the POC and of the POC in the complex were not part of the FF. This shows how the binding energy was a good choice for a parameter that would also affect other features such as the cavity size.

**Table 1:** The fitness values and properties of the final cages.

Cage	FF	*E*_binding_	*D*_complex_	*A*_complex_	*D*_cage_	*A*_cage_
**C1**	15.732	−265.5	10.4	0.03	11.6	0.13
**C2**	14.047	−268.7	10.2	0.05	10.3	0.14
**C3**	4.136	−227.6	10.2	0.06	10.5	0.07
**C4**	3.683	−223.6	10.2	0.02	10.5	0.06
**C5**	3.180	−219.6	10.2	0.02	10.4	0.05
**C6**	2.973	−217.7	10.3	0.01	11.2	0.02
**C7**	2.435	−223.7	10.1	0.05	9.9	0.18
**C8**	1.417	−194.8	10.4	0.03	10.8	0.09
**C9**	1.361	−193.0	10.1	0.03	10.0	0.03
**C10**	1.211	−188.7	10.1	0.03	10.2	0.02
**C11**	1.103	−185.2	10.1	0.02	10.2	0.03
**C12**	1.074	−204.2	9.9	0.08	9.3	0.23
**C13**	1.042	−190.7	10.1	0.12	10.0	0.13
**C14**	1.025	−218.5	10.2	0.05	10.7	0.27
**C15**	0.894	−200.5	10.1	0.16	10.2	0.14
**C16**	0.664	−164.7	9.9	0.06	9.4	0.04
**C17**	0.640	−169.4	9.7	0.13	8.9	0.06
**C18**	0.634	−172.9	10.6	0.04	11.1	0.23
**C19**	0.630	−163.6	10.6	0.09	11.5	0.04
**C20**	0.628	−195.5	10.4	0.18	10.8	0.03

*D*_complex_ is the cavity diameter of the cage extracted from the complex and *D*_cage_ is the cavity diameter of the empty cage. Energies are in kJ mol^−1^ and distances in Å

**Fig. 8:**
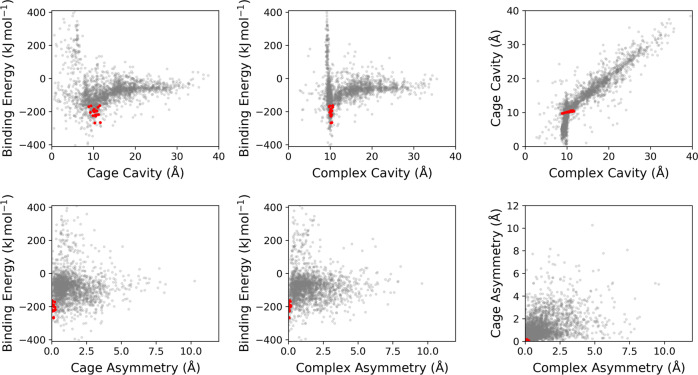
The position of the top candidates in the property space explored. The features of the final cages (red) in comparison to the features of the entire database (grey).

In Fig. 8 and Supplementary Figs. 20–23, we show where the top results are located in terms of properties relative to the entire database. The identified solutions are highly localised, especially for the features that were part of the FF. This is somewhat equivalent to finding the global minimum on the chemical hyperspace, although here we do not aim at a global minimum, rather finding good solutions for POC C_60_ encapsulants. The results are especially promising as some of the building blocks that repeatedly occur in the top candidates have been previously used to synthesise cages. For example, Ding et al. synthesised a [4 + 6] triazine cage with cyclohexylediamine in 2015^37^; the triazine building block used in this example is similar to **node15** that occurs in POCs **C3**, **C4**, **C5** and **C14**, differing only in the number of nitrogen substitutions in the central heteroatomic benzene ring. Further, **node16** in **C17** was previously used to synthesise a [4 + 6] POC called **CC5** when combined with cyclopentyldiamine^38^, and in our prediction, **linker33** is a substituted cyclopentyldiamine. Most recently, truxene building blocks, structurally similar to **node38** in **C1** and nine other cages, have been used to synthesise [4 + 6] POCs with ethylenediamine^39^ and cyclohexyldiamine^40^. However, it has been reported that a [2 + 3] capsule is actually formed with cyclohexyldiamine when using a truxene containing the same trialdehyde substitution pattern as in **C1** and the other examples^40^, rather than the targeted [4 + 6] cages here. Although this does not mean that the [4 + 6] complexes would not necessarily be formed in the presence of C_60_ instead if a templating effect was to occur, rather than relying on diffusion of the C_60_ into a pre-formed cage cavity.

### Design principles for POC encapsulants of C_60_

In addition to the set of specific target cages for POC encapsulation of C_60_ and the development of a FF that can be applied to search much larger databases of building blocks, we can identify the ideal features of any POC for that task. Firstly, the optimal cavity size is in the range of 9.3–11.6 Å. If considering a [4 + 6] imine cage, the tri-topic aldehyde should be roughly  ~16 Å in diameter, and the di-topic amine should have the amine functionality on neighbouring carbon atoms. Many of the precursors used here could be simplified towards alternatives that were successfully used to synthesise cages in the past. However, the best molecule, **C1**, has the amine functionality in greater separation (not on neighbouring carbon atoms), thus larger aldehydes should be considered for combination with this diamine. To our knowledge, there are currently no studies in the literature of C_60_ encapsulation in [4 + 6] imine cages. However, the experimental examples of cages, listed in the previous section, are structurally similar to the presented set of cages here.

## Discussion

We have shown a computational approach using our developed evolutionary algorithm for the discovery of POCs as potential C_60_ fullerene encapsulants. The whole process from the choice of the database of precursors, FF construction, the assembly of a database for parameterisation, and the analysis of the results provided insights into each of these steps. The presented methodology can be used in place of experimental serendipitous discovery, or for the opposite, to facilitate and improve rational design of new functional materials by providing insightful structure-property relationships. The EA and the constructed FF were found to efficiently identify promising candidates for experimental consideration to find new C_60_ encapsulants. More importantly, the same setup could be used for larger databases of building blocks, for any encapsulations, and the discussed parameterisation approaches conducted for extension to other properties and/or molecular material systems.

Design principles can be formulated from the results. The aldehyde building blocks should be fairly planar, with a circular diameter in the range of 14.1–17.0 Å. The amine linkers with amine functional groups on the neighbouring carbons result in the most promising cages. However, larger linkers such as 1,10-phenanthroline-2,9-diamine, which is in our top candidate (**C1**), should also be considered. In all cases, the building blocks have a small number of rotatable bonds, and a high number of aromatic rings. The combination of the building block and linker should result in a cavity size of ~10 Å in diameter.

If we consider a hypothetical database of 30,000 di-topic linkers and 10,000 tri-topic nodes, and if we extend the possible topologies ([2 + 3], [4 + 6], [8 + 12]), the resulting combination of all possibilities would reach 900 million imine cages. Extending our dynamic covalent reaction chemistries to include reactions beyond imine condensation, and to precursors with different numbers of reactive end groups, would quickly result in billions of possibilities. The use of an effectively parameterised EA, as we have presented here based on a parameterisation using just 3870 cages, a tiny fraction of the potential search space, to effectively explore this search space is therefore necessary, as it is not possible to conduct a brute force search of billions of possible POCs. Our approach can also be easily modified to target other properties of molecular materials, such as the likelihood of guest diffusion through a pore window, the size and shape of the host molecule, but also other properties, such as optoelectronic properties in organic electronics.

## Methods

### Cage assembly

The POCs were assembled with our *stk* software by placing the nodes on the vertices and linkers on the edges of a template tetrahedral geometry^13^. Through the selection of high symmetry precursors and this symmetrical topology, we are targeting symmetrical assemblies, which can be anticipated to help increase the chance of synthetic realisation and simplify the number of structural possibilities. The assembly process of the related C_60_ complex uses a new function in *stk*, where the C_60_ is placed at the centre of the template tetrahedral geometry at the very beginning of the assembly process before any geometry optimisation of the POCs. The following procedure for finding the lowest energy POC conformer was performed on the empty cages and their complexes using the OPLS3 force field^41^ in Schrödinger LLC’s MacroModel (Release 2016-2). We have previously found that OPLS3 reproduces well the structure and energetics of porous imine cages^7,9^. Firstly, a geometry optimisation was performed with a convergence criterion of a gradient change smaller than 0.05 with all bonds, apart from those created during the assembly step (imine bonds), restricted during the geometry optimisation. This is followed by a Molecular Dynamics (MD) run at 700 K and timestep of 1 fs to explore the conformational landscape for the molecule. A 10 ps equilibration is followed by a 200 ps production run that is sampled every 10 ps, with each sampled structure being fully geometry optimised. The configuration with the lowest energy at this stage is selected and evaluated with the FF.

### FF parameterisation

The cavity diameter (*D*) and the window diameters used to calculate the asymmetry of a cage (*A*_complex_) and its complex (*A*_cage_) were obtained with *pywindow* (implemented as a part of the *stk* software). The asymmetry (*A*) is defined as the difference between all the window diameters in a cage. First, the window diameters are calculated. Then, the asymmetry is calculated as a sum of the differences in all window diameters. The more comparable the window diameters are, the lower the asymmetry of a cage. We have previously found low asymmetry scores to be a good indication of the high structural symmetry observed in shape-persistent and non-collapsed cages of a tetrahedral topology, built from high symmetry precursors^14^. Tri-topic nodes that are usually at least *C*3_*v*_ symmetry are likely to form highly symmetric assemblies when connected into a tetrahedral topology, unless the assembly is strained. Avoiding highly strained assemblies should ideally increase the likelihood that the hypothetical cages predicted can be realised in the laboratory. The binding energy is calculated with the formula:

3 $${E}_{\mathrm{binding}}={E}_{\mathrm{complex}}-{E}_{\mathrm{cage}}-{E}_{{\mathrm{C}}_{60}}$$

where the total energy (${E}_{{\mathrm{C}}_{60}}$) of an isolated C_60_ molecule is obtained through finding their lowest energy conformers.

In Eq. (1), *E*_binding_ and *A*_complex_ have their values normalised to ensure that all the parameters are positive, as for example the *E*_binding_ can be positive or negative. For each parameter, the lowest value in the population is found and then this value is added to this parameter of the entire population, ensuring all the values are greater than zero. Then, all the values are normalised by dividing them with the mean value of a given parameter within the population. Each parameter can then be multiplied by a constant or raised to some power. The final FF is the sum of all the parameters raised to the power of  −1.

In the EA calculation, the FF is being minimised, so a set of 10 candidates with the lowest fitness value was taken for each parameter set up and the solutions rated. A total rating of the top 10 candidates (*R*_10_) was calculated with a new equation based on the sum of unscaled properties for *E*_binding_ and *A*_complex_ for each member (*i*) of the set:

4 $${R}_{10}=\sum _{i=1}^{10}\frac{R{({E}_{\mathrm{binding}})}_{i}+R{({A}_{\mathrm{complex}})}_{i}}{2}\qquad$$

where $R{({E}_{\mathrm{binding}})}_{i}$ for *i*th complex was calculated using the following formula:

5 $$R{({E}_{\mathrm{binding}})}_{i}=\left\{\begin{array}{ll}1,&\,\text{if}\,\ {E}_{\mathrm{binding}} \, > \, {0}\ \mathrm{kJ}\ {\mathrm{mol}}^{-1}\\ 1,& \, {\text{if}} \,\ {E}_{\mathrm{binding}} \, < \, {-404}\ \mathrm{kJ}\ {\mathrm{mol}}^{-1}\\ 1-\frac{{E}_{\mathrm{binding}}}{-404\ \ \mathrm{{mol}}^{-1}},&\, {\text{otherwise}}\end{array}\right.$$

A positive binding energy, i.e., a lack of binding affinity, is penalised by adding 1 to the *R*_10_ value. At the same time, binding energies lower than  −404 kJ mol^−1^ also result in a penalty of 1. The reason for this is explained in the analysis of the results of the assembled cages and relates to the fact that some forcefield binding energies are unreasonable. The strongest binding energy among the assembled complexes, with the exception of the unreasonable values, was calculated to be  ~−404 kJ mol^−1^. Therefore, the complexes with *E*_binding_ between  −404 and 0 kJ mol^−1^ are assigned a value from 0 to 1, depending on how strong the binding affinity is, resulting in a decreasing penalty for binding energies up to  −404 kJ mol^−1^. In our case here, because we had pre-run our small database, we knew that  −404 kJ mol^−1^ was the lower limit on acceptable binding energies. When moving this study to a larger search space, with unknown binding energies, we would suggest a lower limit of  −780 kJ mol^−1^, which is just below the theoretical limit for a C_60_ complex binding energy reported by Grimme et al.^36^.

The $R{({A}_{\mathrm{complex}})}_{i}$ is calculated with the following formula:

6 $$R{({A}_{\mathrm{complex}})}_{i}=\frac{{A}_{\mathrm{complex}}}{11.864 \AA}$$

where the lower the asymmetry of the cage in the complex, the lower the penalty. The asymmetry parameter is treated here as a proxy for low-strained structures that are more chemically feasible. The value of 11.864 is the highest (worst) asymmetry value in the whole database of 3870 cages. The results were then used to generate 2D heat-maps that allowed us to find the parameters *a*, *b*, *c* and *d* that yield the set of ten best candidates out of the population most effectively.

### Evolutionary algorithm calculations

Overall, the implementation, for example of initialisation, mutation and crossover is as described in our previous work^14^. The selection function used to choose members for the next generation was a roulette wheel, where the probability of selecting a member is proportional to its fitness value. The EA steps are as follows:

1. First the initial population of 20 diverse cages is generated. Random nodes and linkers are chosen and the cages and the corresponding C_60_ complexes generated. This way a set of 20 random cages is generated. In each of the 5 EA runs, a different random initial population was generated.
2. The crossover operation is then applied to a random pair of cages, exchanging building blocks between the pair to result in two offspring molecules. The crossover operation was performed 7 times in each generation.
3. The mutation operation is applied 10 times in each generation, with the fittest population member always undergoing a mutation. The remaining 9 mutation candidates were chosen using roulette wheel probability. A cage is chosen at random and then its’ fitness is compared to a randomly generated value between 0 and 1. If the fitness of the candidate is greater than the randomly generated number, then the cage undergoes one of the mutation functions. This is repeated until 9 cages are mutated. There were four mutations applied with equal probability; exchange of the linker to a similar one (the linker with the closest Dice similarity to that being exchanged), the exchange of the node to a similar one (the node with the closest Dice similarity to that being exchanged), the exchange of the linker to a random one, and the exchange of the node to a random one. This provides an excellent balance between small and large steps across the chemical search space^14^.
4. This results in a total of 44 cages, 20 coming from the current generation, 14 from crossover and 10 from mutation. From these, 20 are chosen using the roulette wheel, to create the next generation. The fittest candidate always proceeds to the next generation unchanged, equivalent to elitism.
5. The whole process is repeated for 100 generations.

The five EA calculations resulted in five final populations of 20 candidates each. These were then combined into a single population and the duplicates were removed. The resulting population consisted of 53 unique members. The fitness of the members of the final population was re-evaluated with the FF from Eq. (2) and the candidates ranked in ascending order.

## Supplementary information

Supplementary Information

## Data Availability

Datasets analysed are available at 10.14469/hpc/6054 and any further data is available on reasonable request from the corresponding author.

## Code availability

The software used here is a development of *stk*, which is available on github at github.com/JelfsMaterialsGroup/stk. For additional features of the software, contact the corresponding author.
