# Supplementary material for: Computational discovery of molecular C60 encapsulants with an evolutionary algorithm
Source: Commun Chem. 2020 Jan 22;3:10. doi: 10.1038/s42004-020-0255-8 (PMC9814092; doi:10.1038/s42004-020-0255-8)
Supplement: Supplementary file 1 — Supplementary Information [file 42004_2020_255_MOESM1_ESM.pdf]

Supporting Information: Computational Discovery of Molecular C_{60} Encapsulants Using an Evolutionary Algorithm

Marcin Miklitz,[†] Lukas Turcani,[†] Rebecca L. Greenaway,[‡] and Kim E. Jelfs[*,†]

†*Department of Chemistry, Molecular Sciences Research Hub, White City Campus, Imperial College London, Wood Lane, London, W12 0BZ, United Kingdom*

‡*Department of Chemistry and Materials Innovation Factory, University of Liverpool, 51 Oxford Street, Liverpool L7 3NY, United Kingdom*

E-mail: k.jelfs@imperial.ac.uk

Phone: +44 (0)20 7594 3438

Contents

1 Precursor library 3

2 Analysis of the assembled cages and complexes 7

3 Analysis of cage evolution 10

4 Feature analysis of the top 20 C_{60} complexes 21

1 Precursor library

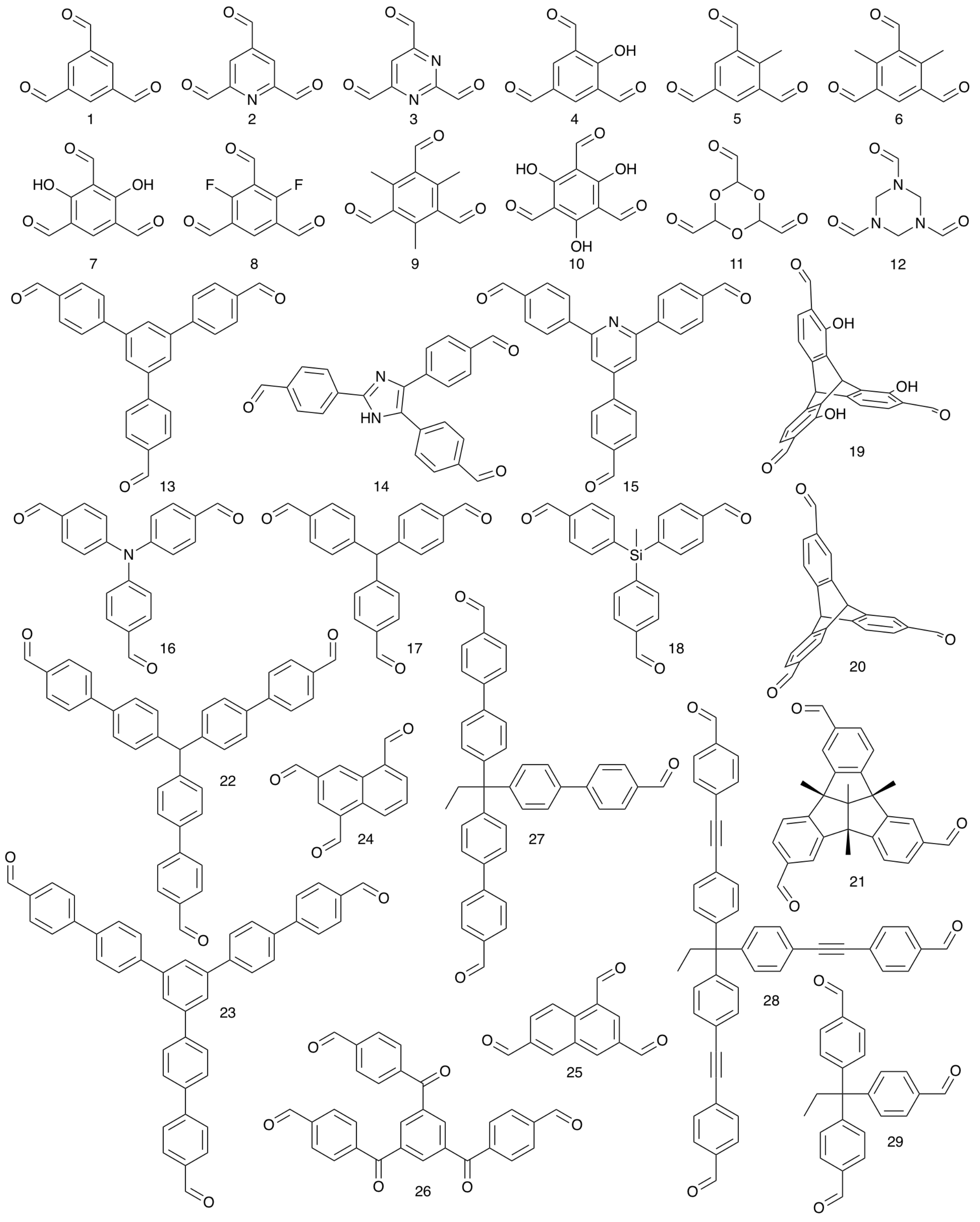

Supplementary Figure 1: The trialdehyde nodes used in the assembly of the POCs - part 1.

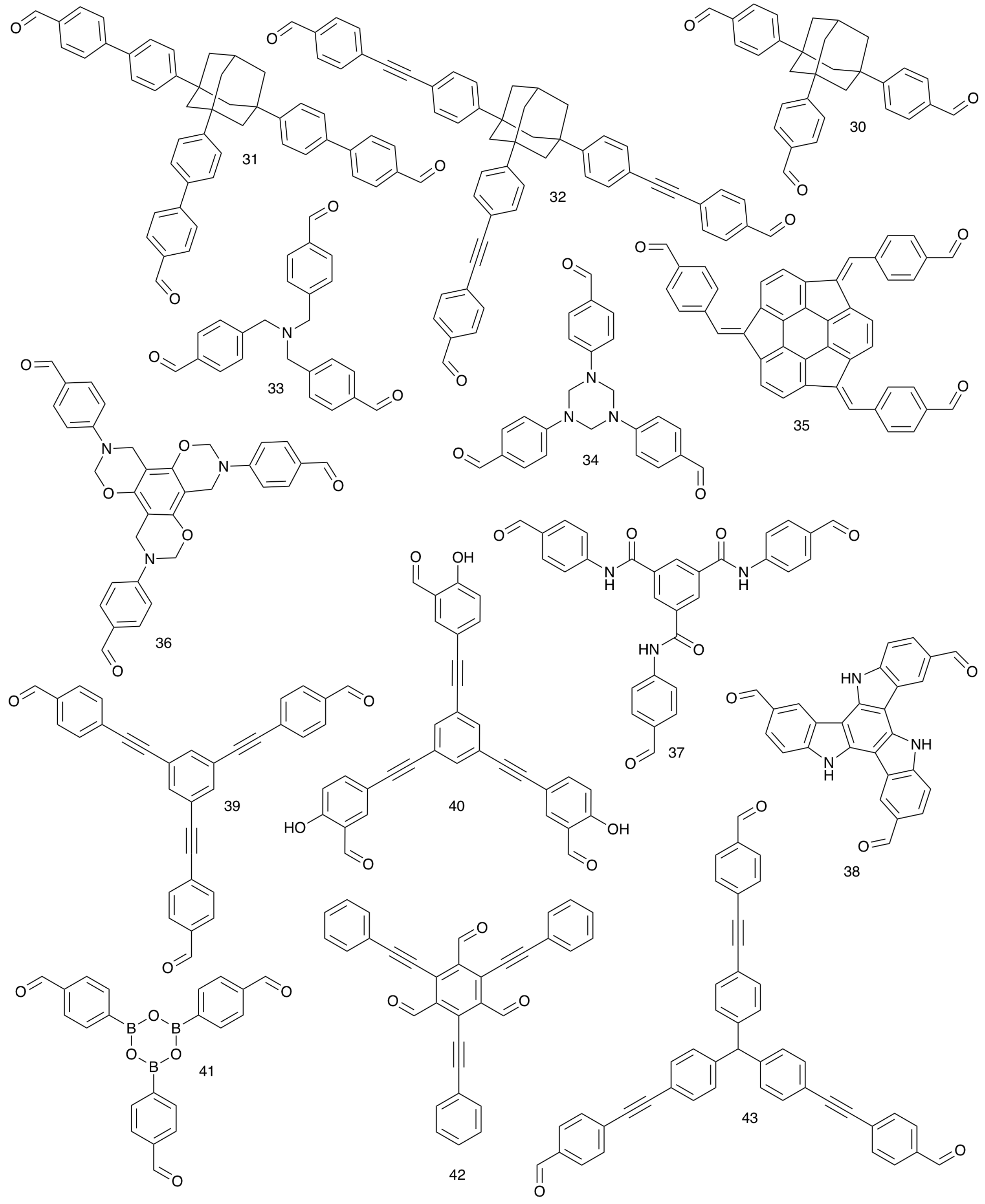

Supplementary Figure 2: The trialdehyde nodesused in the assembly of POCs - part 2.

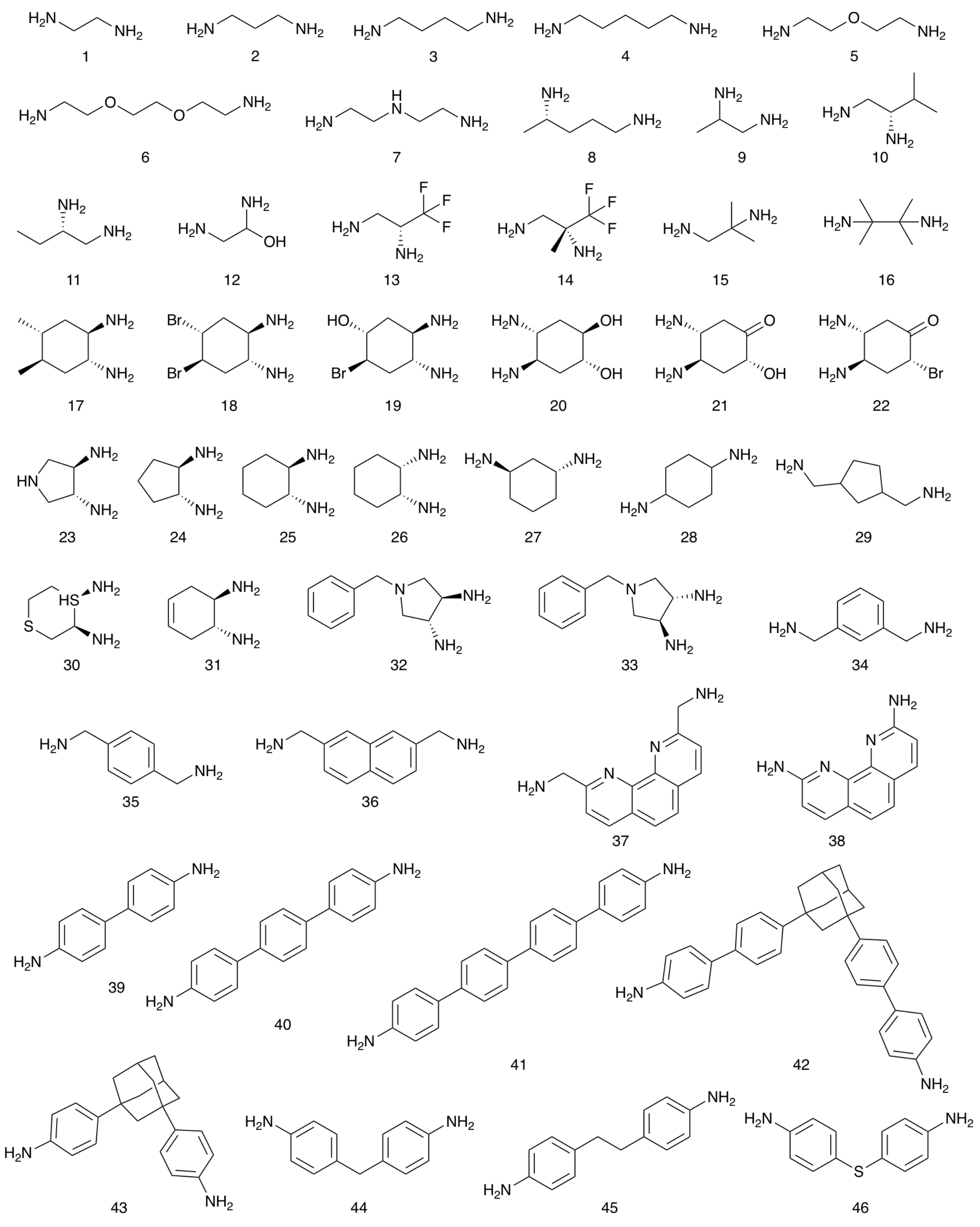

Supplementary Figure 3: The diamine linkers used in the assembly of POCs - part 1.

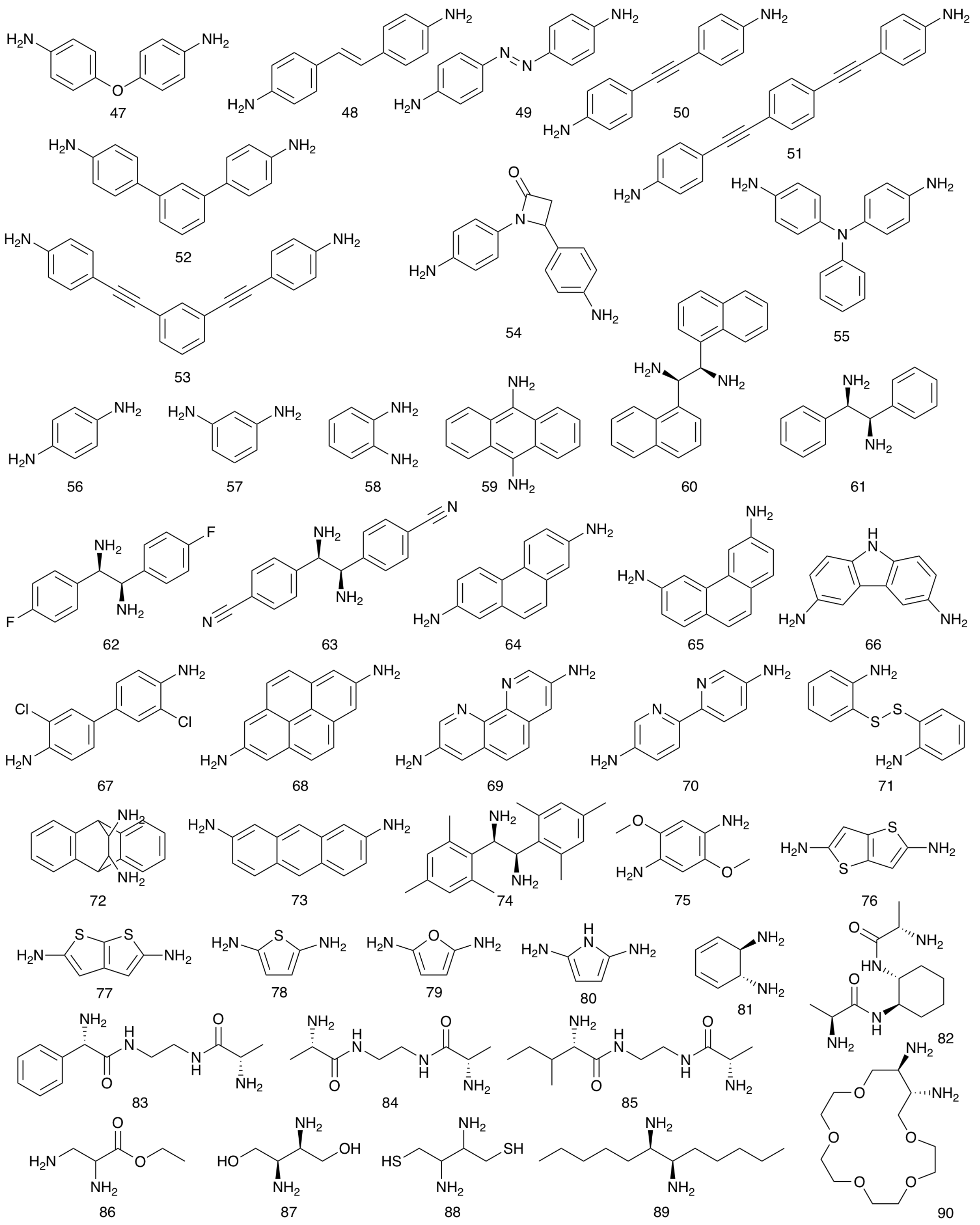

Supplementary Figure 4: The diamine linkers used in the assembly of POCs - part 2.

2 Analysis of the assembled cages and complexes

Presented below is the analysis of the assembled database of cages and the corresponding C_{60} complexes. Various properties calculated as input for the fitness function include: the binding energy in the complexes, the cavity size of cage in complex, the asymmetry of cage in the complex, the strain of cage in the complex, the cavity size of an empty cage, the asymmetry of an empty cage and the strain of an empty cage. The complexes were divided into three sets. These with binding energies greater than 0 kJ mol^{-1} (coloured blue), the cages with binding energies within the range of -404 and 0 kJ mol^{-1} (coloured green), and the complexes with binding energies well below -404 kJ mol^{-1} (coloured red).

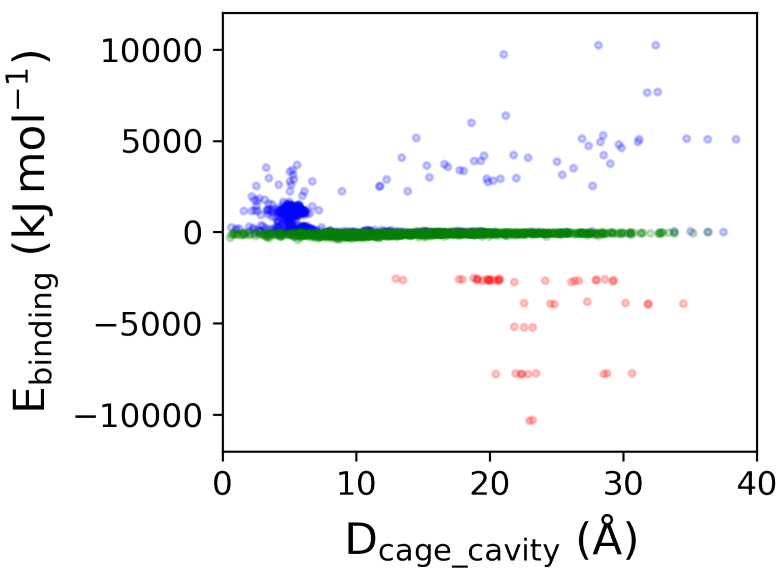

Supplementary Figure 5: The binding energy of the complexes and the cavity size of the corresponding empty cages. The cages have been divided based on their binding energies into three sets; those with binding energies greater than 0 kJ mol^{-1} (blue), within the range of -404 and 0 kJ mol^{-1} (green), and below -404 kJ mol^{-1} (red).

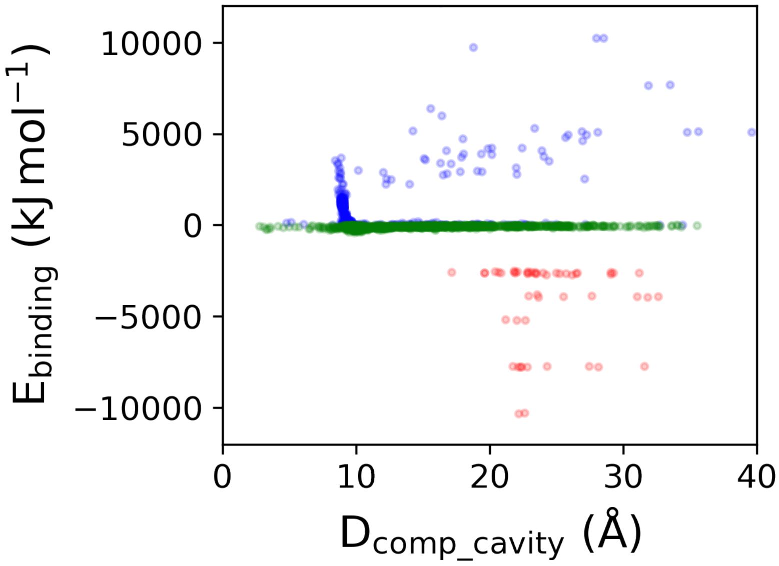

Supplementary Figure 6: The binding energy in the complexes and the cavity size of the cage after C_{60} has been removed from the complex. The cages have been divided based on their binding energies into three sets; those with binding energies greater than 0 kJ mol^{-1} (blue), within the range of -404 and 0 kJ mol^{-1} (green), and below -404 kJ mol^{-1} (red).

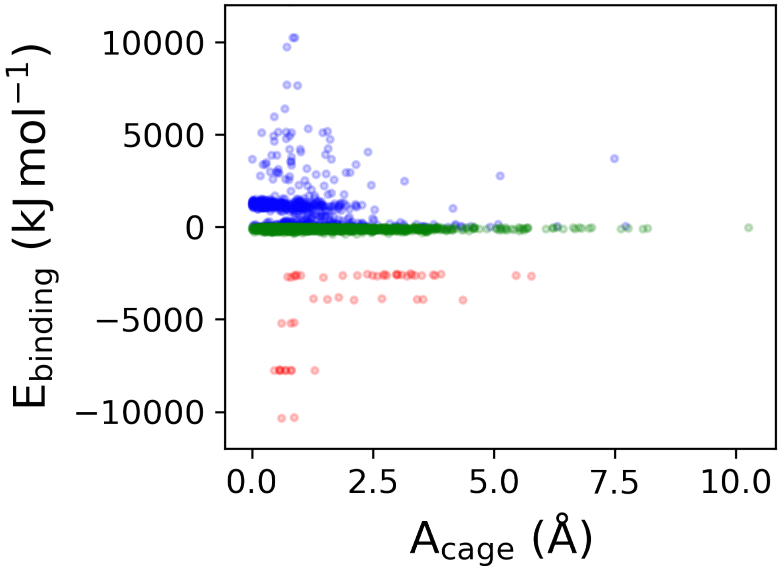

Supplementary Figure 7: The binding energy in the complexes and the asymmetry of the cage in the corresponding empty cages. The cages have been divided based on their binding energies into three sets; those with binding energies greater than 0 kJ mol^{-1} (blue), within the range of -404 and 0 kJ mol^{-1} (green), and below -404 kJ mol^{-1} (red).

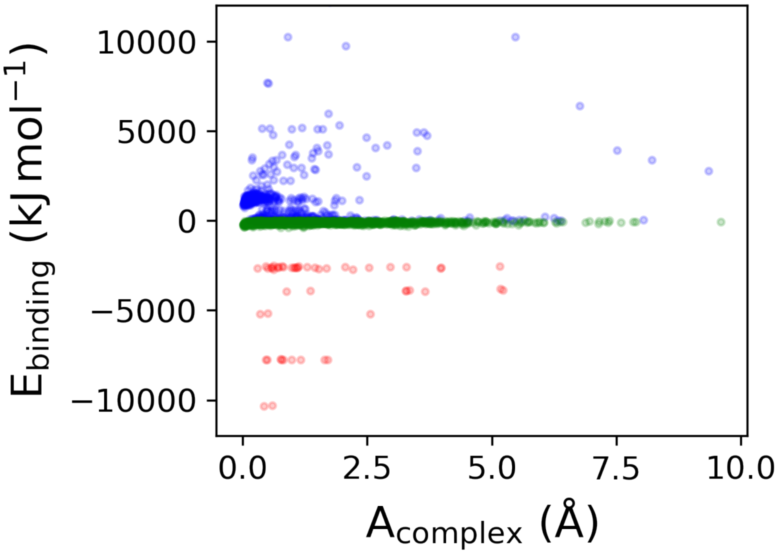

Supplementary Figure 8: The binding energy in the complexes and the asymmetry of the cage after C_{60} removed from the complex. The cages have been divided based on their binding energies into three sets; those with binding energies greater than 0 kJ mol^{-1} (blue), within the range of -404 and 0 kJ mol^{-1} (green), and below -404 kJ mol^{-1} (red).

3 Analysis of cage evolution

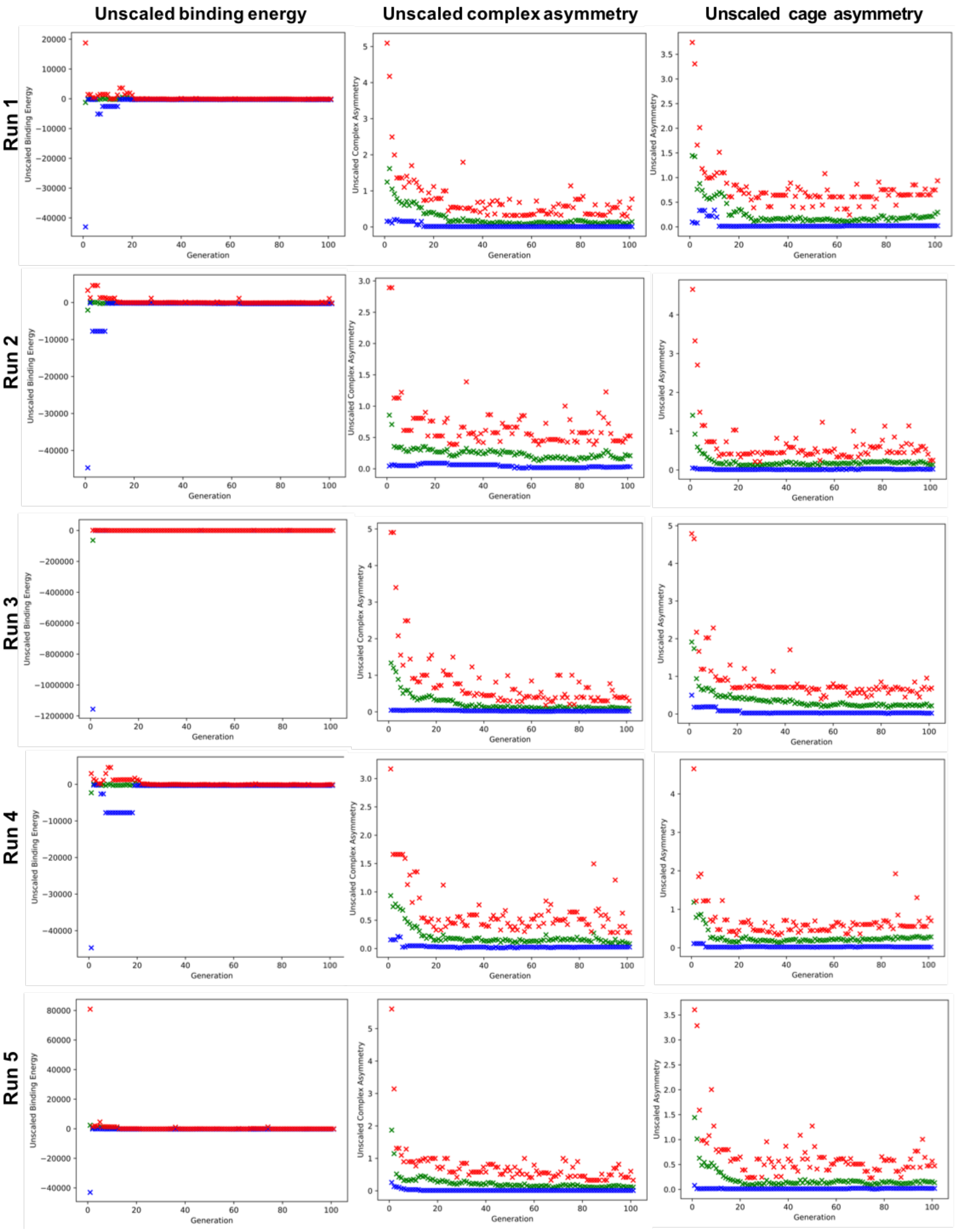

Supplementary Figure 9: Plots showing the values of the maximum (red), minimum (blue) and mean (green) of each of the unscaled components of the fitness function over the evolution of the five runs.

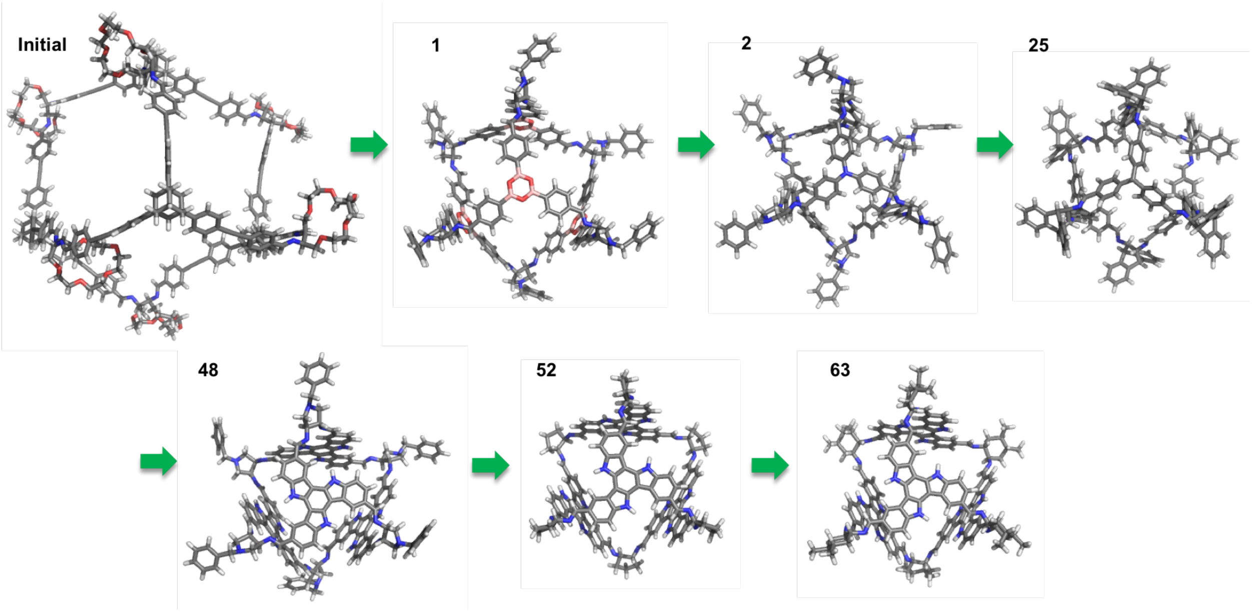

Supplementary Figure 10: The top performing candidates from run 2 at different stages of the evolution. The generation number where each structure becomes the top performing is labelled.

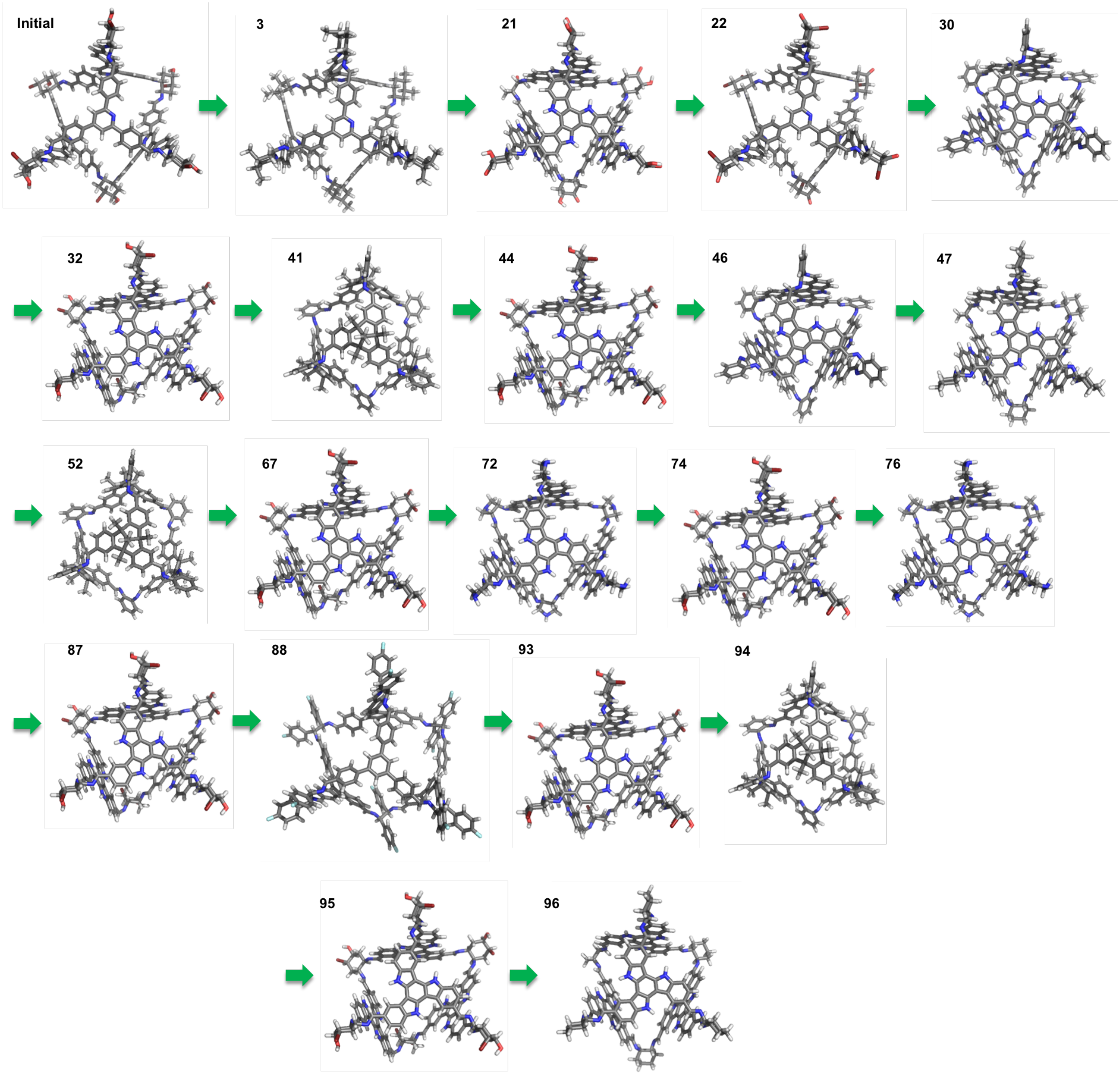

Supplementary Figure 11: The top performing candidates from run 3 at different stages of the evolution. The generation number where each structure becomes the top performing is labelled.

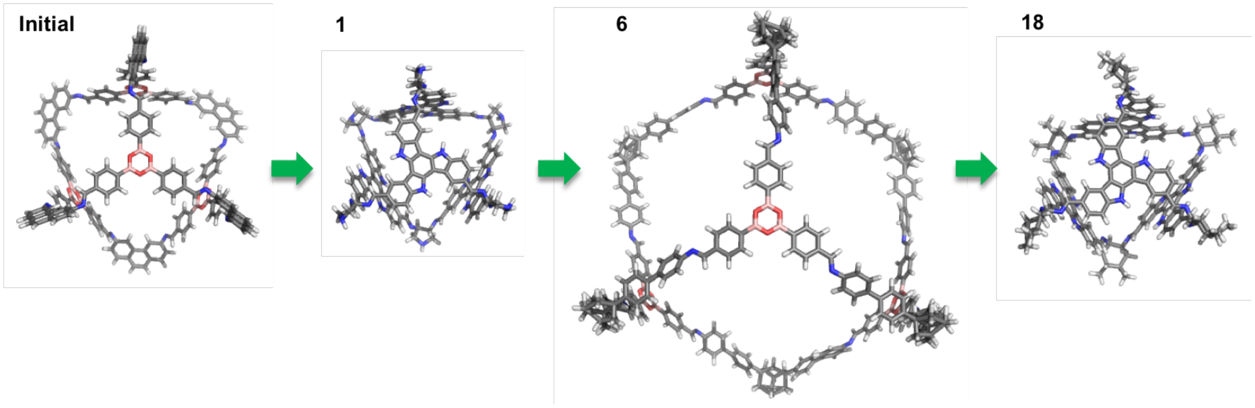

Supplementary Figure 12: The top performing candidates from run 4 at different stages of the evolution. The generation number where each structure becomes the top performing is labelled.

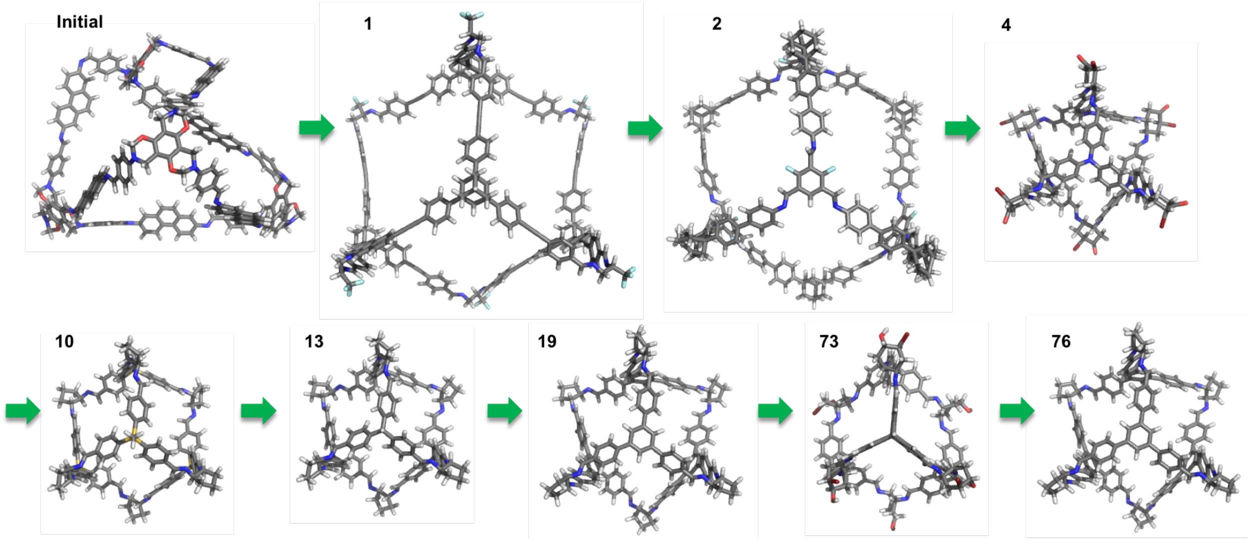

Supplementary Figure 13: The top performing candidates from run 5 at different stages of the evolution. The generation number where each structure becomes the top performing is labelled.

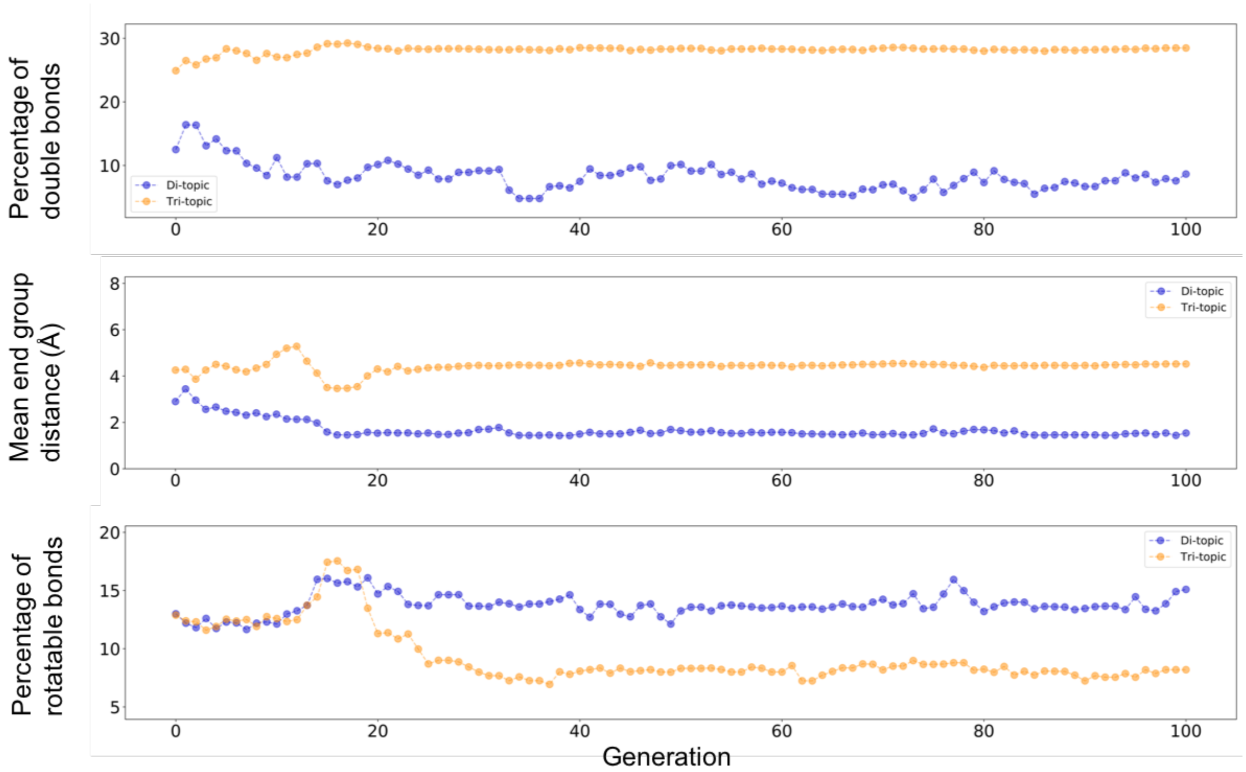

Supplementary Figure 14: Analysis of the average molecular properties of the di-topic linkers (blue) and tri-topic nodes (orange) for the individuals in each population of run 1. For each EA run, we investigated (top) the change in the percentage of double bonds, (middle) mean distance between reactive end groups and (bottom) percentage of double bonds.

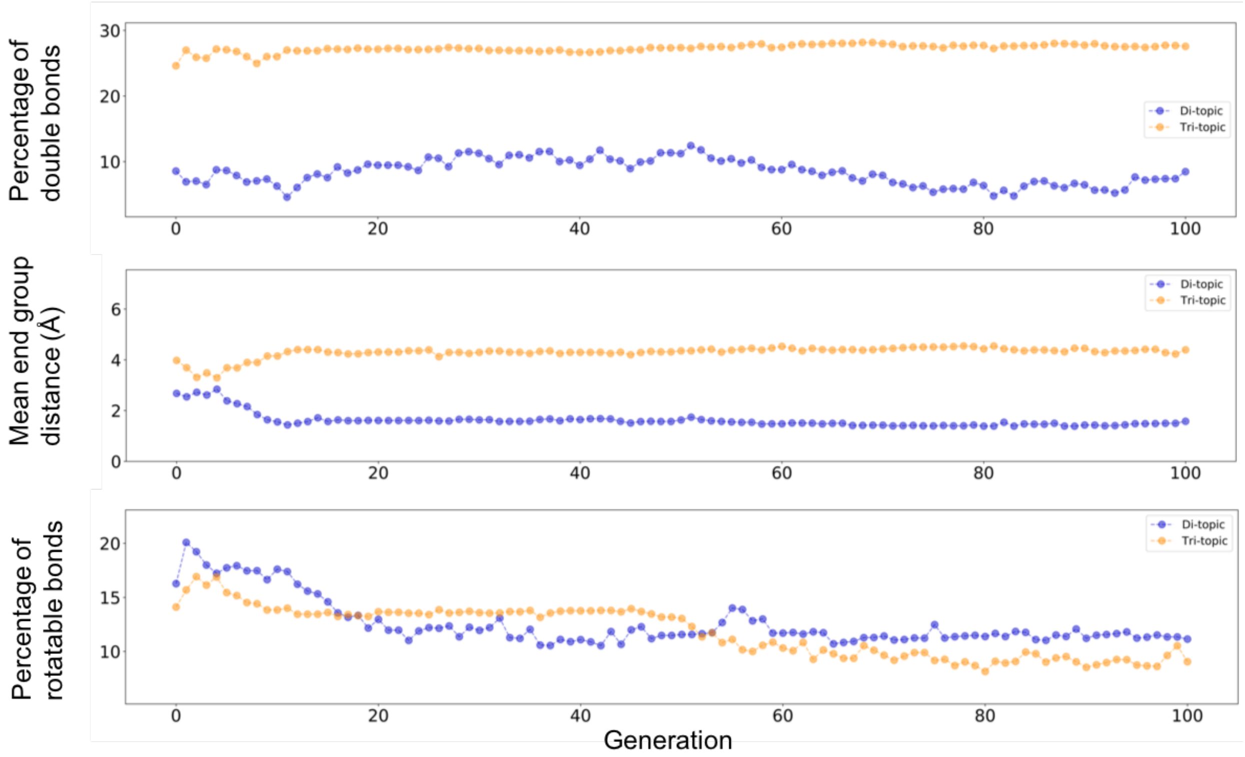

Supplementary Figure 15: Analysis of the average molecular properties of the di-topic linkers (blue) and tri-topic nodes (orange) for the individuals in each population of run 2. For each EA run, we investigated (top) the change in the percentage of double bonds, (middle) mean distance between reactive end groups and (bottom) percentage of double bonds.

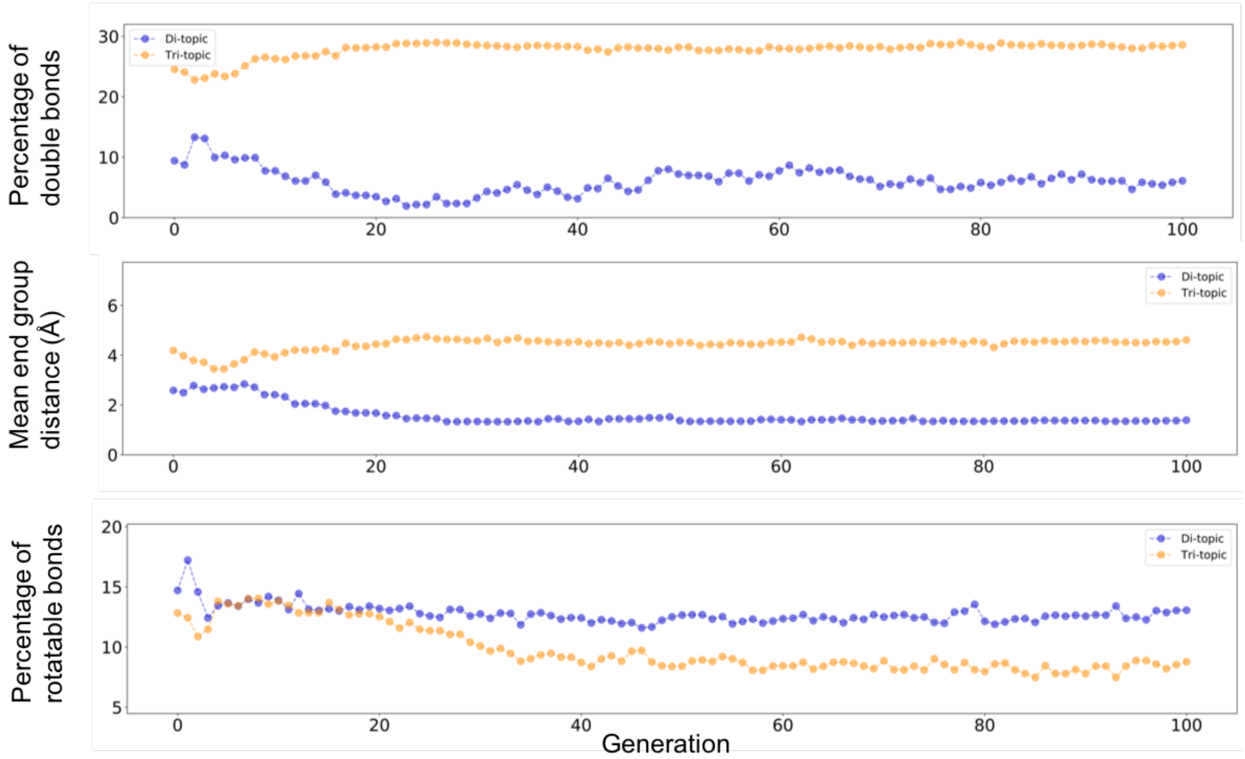

Supplementary Figure 16: Analysis of the average molecular properties of the di-topic linkers (blue) and tri-topic nodes (orange) for the individuals in each population of run 3. For each EA run, we investigated (top) the change in the percentage of double bonds, (middle) mean distance between reactive end groups and (bottom) percentage of double bonds.

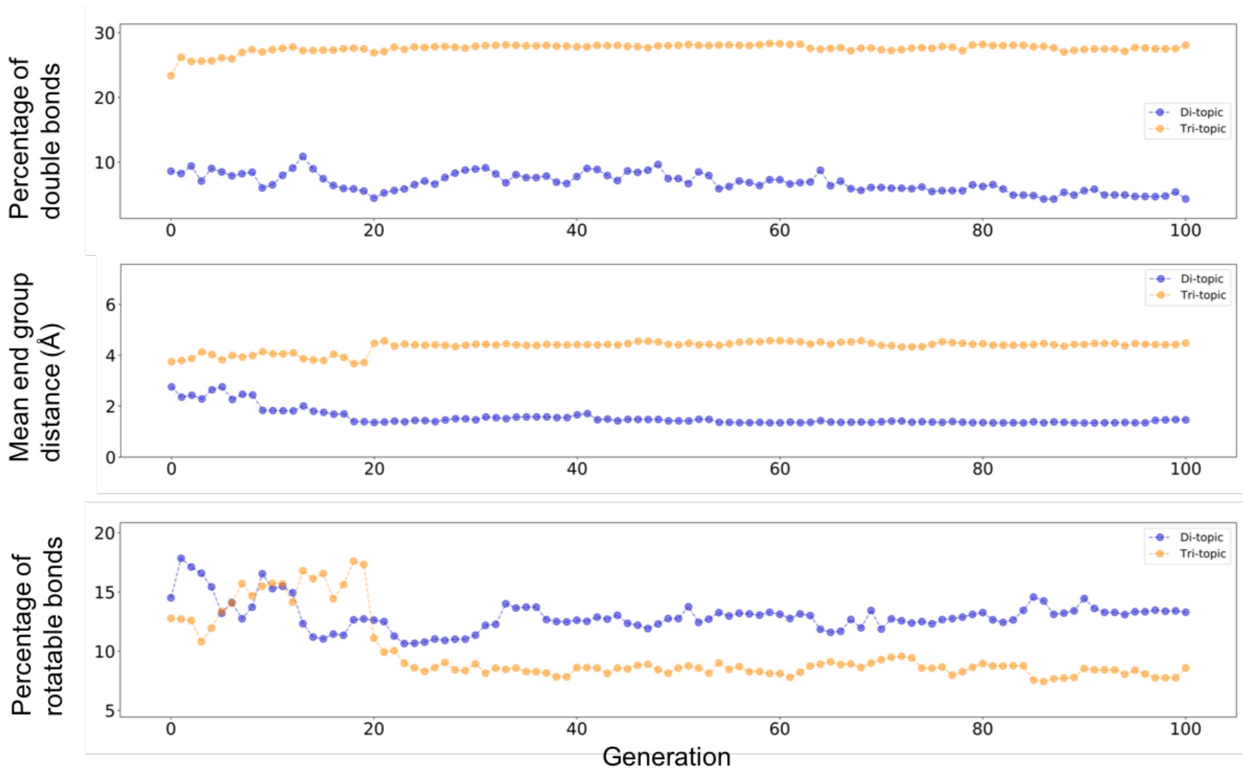

Supplementary Figure 17: Analysis of the average molecular properties of the di-topic linkers (blue) and tri-topic nodes (orange) for the individuals in each population of run 4. For each EA run, we investigated (top) the change in the percentage of double bonds, (middle) mean distance between reactive end groups and (bottom) percentage of double bonds.

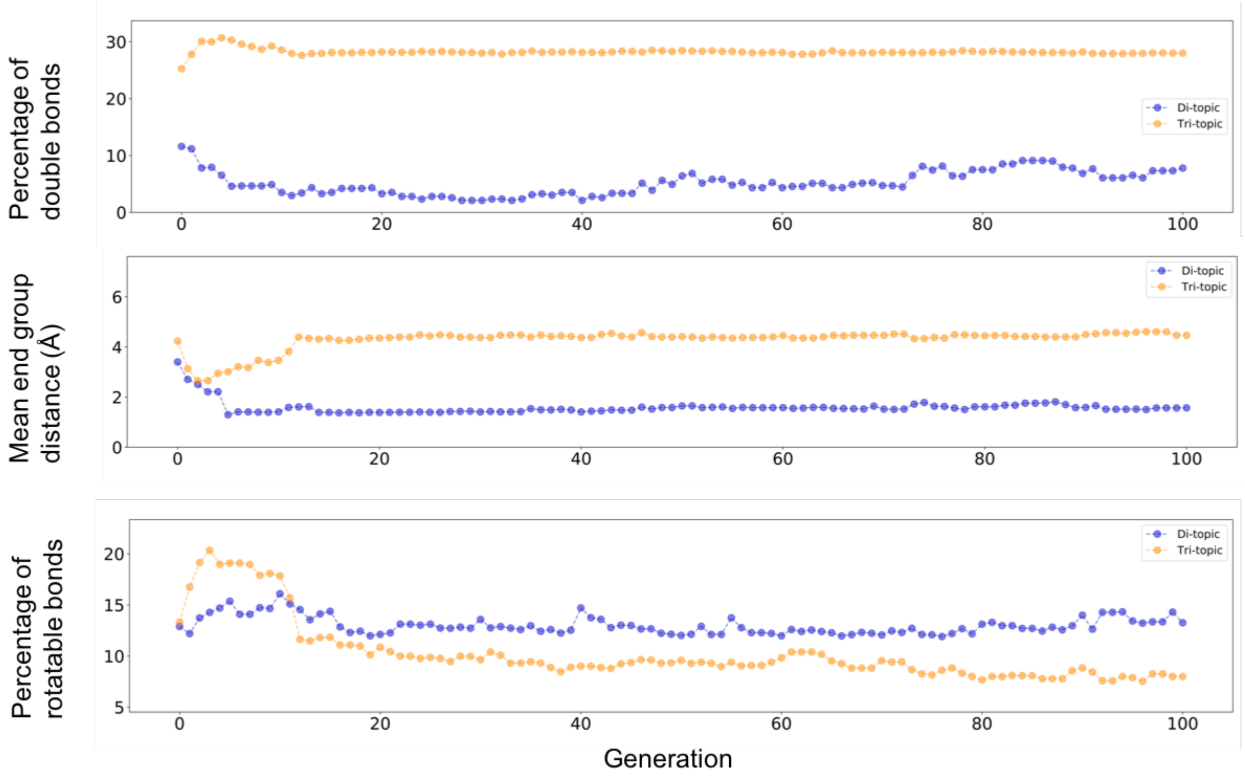

Supplementary Figure 18: Analysis of the average molecular properties of the di-topic linkers (blue) and tri-topic nodes (orange) for the individuals in each population of run 5. For each EA run, we investigated (top) the change in the percentage of double bonds, (middle) mexan distance between reactive end groups and (bottom) percentage of double bonds.

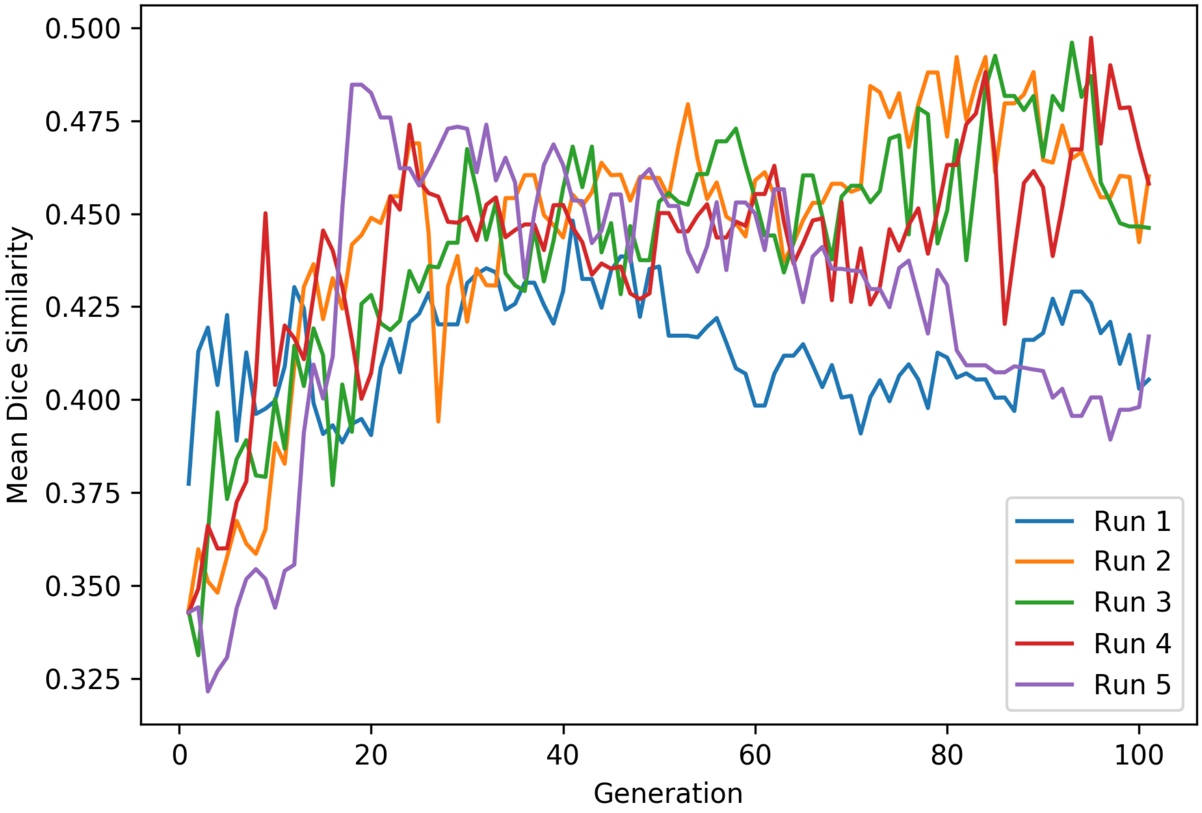

Supplementary Figure 19: Plot showing the evolution of the chemical diversity of the fragments calculated over the generations for the five runs. The diversity is calculated by taking each individual instance of a building block in a generation, calculating the Dice similarity (as implemented in RDKit) with every other building block in that generation, then calculating the mean of those values for each generation.

4 Feature analysis of the top 20 C_{60} complexes

Presented below is the analysis of the 20 most promising cages and their corresponding C_{60} complexes.

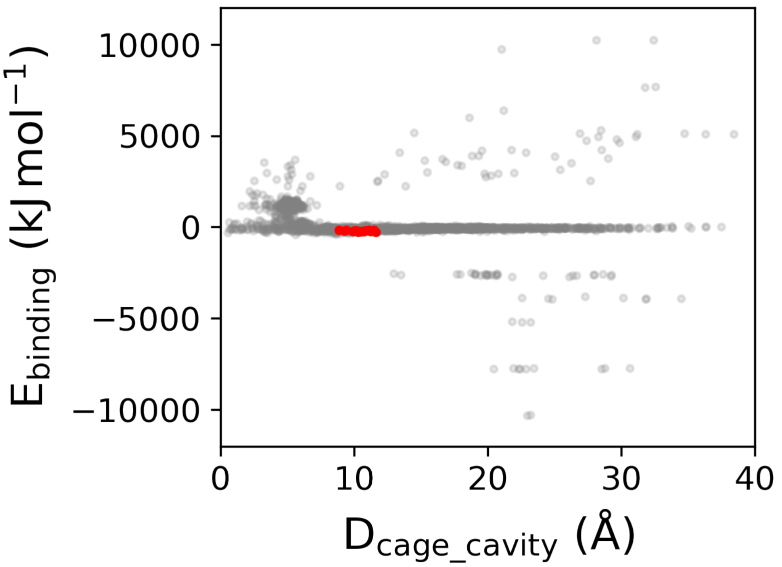

Supplementary Figure 20: The binding energy of the final C_{60} complexes and the pore diameters of the corresponding empty cages. The 20 most promising systems are coloured red, the remainder grey.

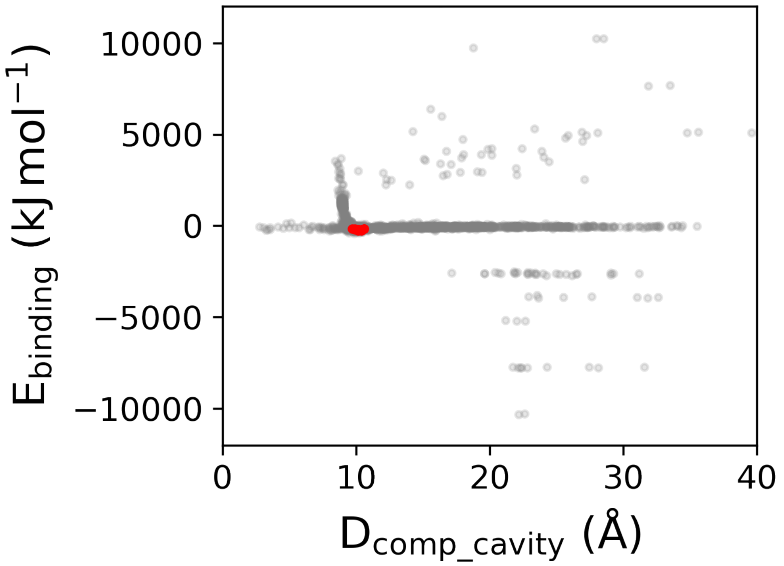

Supplementary Figure 21: The binding energy of the final C_{60} complexes and the pore diameters of the corresponding cages after C_{60} removal. The 20 most promising systems are coloured red, the remainder grey.

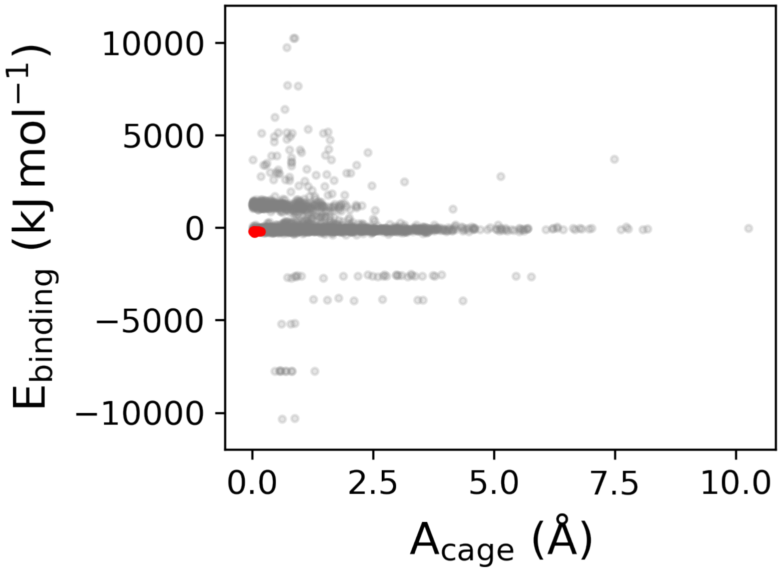

Supplementary Figure 22: The binding energy of the final C_{60} complexes and the asymmetry of the corresponding empty cages. The 20 most promising systems are coloured red, the remainder grey.

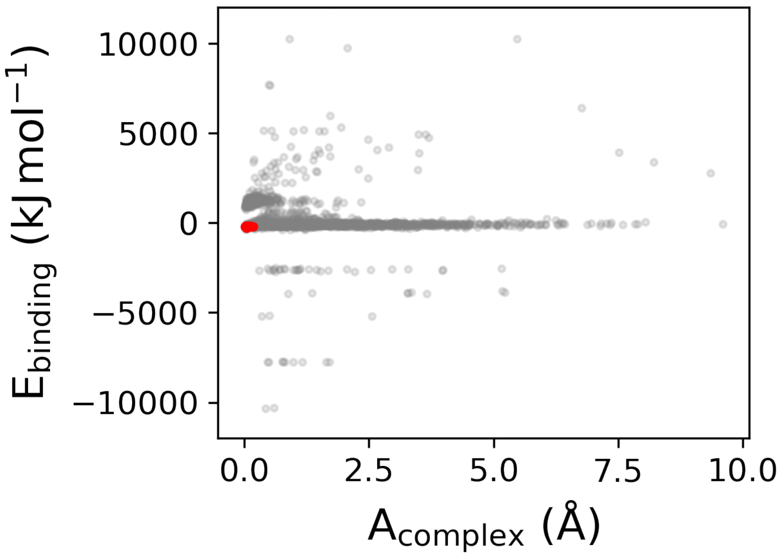

Supplementary Figure 23: The binding energy of the final C_{60} complexes and the asymmetry of the corresponding cages after C_{60} removal. The 20 most promising systems are coloured red, the remainder grey.
